# Diabetes-related distress and associated factors among adult diabetes mellitus patients attending public hospitals in Gedio zone, southern Ethiopia: Mediation analysis

**DOI:** 10.1371/journal.pone.0331655

**Published:** 2025-09-29

**Authors:** Alem Bayable Mersha, Atirsaw Shimekaw, Edom Gebremedhin, Jerusalem Sewalem, Andualem Bayih, Abel Desalegn Demeke, Simret Gebre

**Affiliations:** 1 Department of Nursing, College of Medicine and Health Science, Dilla University, Dilla, Ethiopia; 2 Department of Internal Medicine, College of Medicine and Health Science, Dilla University, Dilla, Ethiopia; 3 Department of Psychiatry, College of Medicine and Health Science, Dilla University, Dilla, Ethiopia; 4 Department of Medical Laboratory, College of Medicine and Health Science, Dilla University, Dilla, Ethiopia; Iran University of Medical Sciences, IRAN, ISLAMIC REPUBLIC OF

## Abstract

**Background:**

Individuals with diabetes might experience distress due to their treatment regimen, interactions with physicians, interpersonal relationships, and emotional well-being. This may lead to lower self-efficacy, which would impair self-treatment adherence and result in poor glycemic control. Understanding and addressing gaps may help individuals with diabetes improve their glycemic control. Therefore, this study aimed to determine the magnitude of diabetes-related distress and associated factors among adult diabetes mellitus patients attending public hospitals in the Gedio zone, Southern Ethiopia.

**Method:**

An institutional-based cross-sectional study was conducted from April to May 2024 at four public hospitals in the Gedio zone. Study participants who met the inclusion criteria were selected using systematic random sampling techniques. Data were collected using the Diabetes Distress Scale (DDS-17). Both bivariable and multivariable ordinal logistic regression were applied. In multi-variable ordinal logistic regression, a P value < 0.05 significant level was used to identify factors of diabetic-related distress. Mediation analyses were conducted using the bootstrapping technique. A variable showing a significant indirect effect was considered as a mediator.

**Results:**

In this study, a total of 506 adult patients with diabetes participated. The overall prevalence for moderate and high levels of diabetes-related distress was 47.04% (95% CI;42.6–51.5) and 41.5% (95% CI; 37.2–45.9), respectively. Variables like never had planned physical activity (adjusted odd ratio, aOR=3.09;95%CI = 1.034–9.28; P = 0.04), poor social support (aOR=3.44;95%CI = 1.42–8.33;P < 0.006), complications (aOR=1.79; 95%CI = 1.13–2.84; P < 0.013) and high blood pressure (aOR= 1.85; 95%CI = 1.12–3.05; P = 0.016) were factors for diabetes related distress. Depression was identified as a partial mediator of the relation between social support and diabetes related distress.

**Conclusion:**

Diabetes-related distress was highly prevalent in diabetes patients. Healthcare providers need to address this by integrating psycho-social care with collaborative medical care.

## Introduction

Diabetes mellitus is a group of long-term metabolic problems indicated by abnormalities in insulin secretion, action, or both [1]. It is a leading cause of morbidity and mortality among people all over the world, and is associated with an increase in unhealthy lifestyles, such as poor eating habits and insufficient physical activity [2–4]. Individuals with long-term diabetes may face not only physical health challenges but also experience negative psychological issues because of the risk of complications and social burden that comes with the disease, which can result in an emotional burden. As a result, managing DM and achieving the desired targeted glycemic control requires additional duties, planning, and self-monitoring [5,6].

The term “diabetes-related distress” (DRD) refers to the worries, concerns, and emotional problems that are associated with managing diabetes, getting support, bearing emotional burdens, and getting access to care [7,8]. When people with diabetes believe that their coping mechanisms are inadequate to manage the threat of their illness, this leads to an emotional burden that is specific to diabetes. Diabetic distress has been divided into four domains, which include emotional, interpersonal, physician, and regimen-related distress [9]. Patients with high DRD are likely to demonstrate poor self-management due to the distress of daily needs of disease management, worries about poor glycemic control, fears about diabetic complications, inadequate support from significant others, stigma, and financial struggles [10,11]. It is widely known that self-care practice and medication adherence determine glycemic control, which is connected to future problems and a lower quality of life [12]. Psychological conditions associated with diabetes can create hypothetically dangerous surroundings that can influence health-seeking behavior, acceptance of the diagnosis, and treatment adherence, which have a significant impact on patients health outcomes. Individuals with DRD are 1.56 times more likely to mortality, and higher risk for morbidity [13]. Different Studies found diabetes distress was significantly correlated to the number of serious complications, including heart disease, stroke, blindness, kidney failure, and lower-limb amputation due to uncontrolled glycemia [2,14–16]. It can also lead to severe medical and psychological outcomes, including reduced physical activity [17], unhealthy eating practices [18], less frequent self-monitoring of blood glucose [19,20], elevated HbA1c [21], more frequent severe hypoglycemia [22], and lowered quality of life [23].

Despite Ethiopia being one of the four nations in sub-Saharan Africa with the highest rates of adult diabetes**,** there are limited studies conducted on the psychological aspect of diabetes in the country [24]. This calls for a change in healthcare provider systems that manage diabetic patients by taking psychological aspects like diabetes-related distress into account [25]. So, this study intended to fill gaps by conducting scientific research on this topic to rule out the magnitude and factors that could affect diabetes-related distress by including individuals with type 1 and type 2 diabetes mellitus.

## Materials and methods

### Study area

The study was conducted at public hospitals in the Gedio zone. Gedio zone is located 359 kilometers south of Addis Ababa and roughly 90 kilometers from Hawassa (capital city of Sidama region). According to the Central Statistical Agency report, the zone has a total population of 847,434 [26]. It has twelve districts or woredas, including Dilla Town, Kochore, Chelelektu Town, Gedeb Town, Chorso, Yirga Chefe Town, Yirga Chefe woreda, Dilla Zuria, Gedeb woreda, Wonago, Bule, and Raphe. There are four public hospitals in the zone, which include one general hospital in Dilla town and three primary hospitals in Bule, Gedeb, and Yirga Chefe weredas.

### Study design and period

An institution-based cross-sectional study was conducted among all diabetic patients who had follow-ups at the outpatient departments of public hospitals in the Gedeo Zone, Southern Ethiopia, from April 20 to May 20, 2024.

### Population

All patients with diabetes mellitus who were receiving follow-up care at the outpatient department of public hospitals in the Gedio zone were considered the source and study population.

### Inclusion and exclusion criteria

This study included adult patients aged 18 years or older who had been diagnosed with either type 1 or type 2 diabetes mellitus and had at least three months of follow-up up but patients who were unable to communicate due to their critical health condition, had a history of psychiatric disorders, and newly diagnosed patients with type 1 or type 2 diabetes were excluded from the study.

### Sample size determination and sampling technique

The sample size was calculated using Epi Info version 7.2. The total sample size was calculated with a 95% confidence level, 80% power, and a 1:1 ratio of unexposed to exposed groups using the double population-proportion formula. The sample size was determined by using the previous study variable of HbA1c (percentage of outcome in the unexposed group = 93.2%, and adjusted odds ratio = 5.49) [27]. So, after adding a 10% non-response rate, the final sample size for this particular study was 521.

The list of respondents, which serves as a sampling frame, was obtained from the follow-up clinic registration logbooks of four public hospitals in the Gedio Zone. After establishing the sampling frames in each hospital, the total sample size was first proportionally allocated according to the number of registered diabetes mellitus patients in each hospital to facilitate the selection of study participants. Then, a systematic random sampling technique with a sampling interval of three was applied to identify the study unit to be included in the study. The people living with diabetes who met the inclusion criteria were recruited for the study until the required sample size was achieved. After proportional allocation, the number of participants recruited from each hospital was 227 from Dilla University Teaching Hospital, 144 from Yergachefe Primary Hospital, 78 from Gedeb Primary Hospital, and 72 from Bule Primary Hospital.

### Operational definition

**Diabetic-related distress (DRD)** is defined as the concerns, worries, and emotional burdens that are negative emotional experiences when managing diabetes. An overall mean score of less than 2 is regarded as little distress, a score ranging from 2 to 2.9 is considered moderate distress, and a score of 3 or higher is considered a high level of distress [9,28].

**Diabetic mellitus (DM)** was defined in this study as a metabolic disorder characterized by the presence of high glucose levels due to poor insulin secretion (Type-1) or poor insulin utilization (Type-2) [29]. In this study, information on the type of diabetes was obtained from patients medical records. The criteria used by each hospital to define poor glycemic control were based on fasting blood glucose (FBG) levels of < 70 mg/dL or ≥ 130 mg/dL.

**Social support** was assessed using the Oslo 3-item social support scale, which assesses the level of support the patient received from family and friends. This scale score ranged from 3 to 14, with scores of 3–8 indicating poor support, 9–11 shows moderate support, and 12–14 was considered as high support [30].

**Substance use** was assessed by using alcohol, smoking, and substance involvement screening test (ASSIST) questionnaires which classified as low risk (0–10 for Alcohol intake and 0–3 for other substances), moderate risk (11–26 for alcohol intake, and 4–26 for other substances), and high risk (greater than 27 for all substance used) [31,32].

**Depression** is a mental disorder characterized by sadness and lack of interest or pleasure in previously rewarding or enjoyable activities, assessed using PHQ-9. A PHQ score of 4–9, 10–19, and greater than 20 suggests mild, moderate, and severe depression, respectively [33].

### Data collection tools

Data was collected using a prepared, structured, and pretested questionnaire developed by reviewing different relevant Literature [34–36] that can address the objective of the study. DDS-17 is a well-validated and widely used tool designed to measure various diabetes related [37–39].Each question has six answer choices: 1(no problem), 2 (slight problem), 3(moderate problem), 4(somewhat serious problem), 5 (a serious problem), and 6 (a very serious problem). The questionnaire contains four domains: emotional burden (contain 5 items: questions 1, 3, 8, 11, and 14); physician-related distress (contain 4 items: questions 2, 4, 9, and 15); regimen-related distress (contain 5 items: questions 5, 6, 10, 12, and 16); and interpersonal related distress (contain 3 items: questions 7, 13, and 17). An overall mean score of less than 2.0 was considered as little to no distress, a score of 2–2.9 as moderate distress, and a score greater than 3 was considered a high level of distress. A four-point Likert scale of PHQ-9 and three items of Oslo social support were used to assess depression and participants level of social support, respectively [30,33]. In addition to this, substance use was assessed using a standardized alcohol, smoking, and substance involvement screening test (ASSIST). The questionnaires for this study consist of four sections (section one: socio-demographic characteristics, section two: diabetes-related distress, section three**:** clinical-related characteristics, and section four: patient-related characteristics). These sections of the questionnaire were either administered through face-to-face interviews for patients who could not read or write, or completed by the patients themselves. Additionally, some clinical data, such as type of diabetes, fasting blood glucose level (FBG), and blood pressure level, were obtained by reviewing patients charts.

### Data collection methods

Data was collected by eight trained BSc degree nurses through face-to-face interviews, self-administered questionnaires, and reviewing the patient charts, and the whole activity of data collection was followed by four supervisors. Written permission to conduct the study was obtained from each hospital before the recruitment of participants for the study. Patients were informed about the study’s purpose and provided written consent before participation. All data collection activities were carried out following patient consent to participate in the study. Interviews were carried out in a quiet room during routine follow-up visits, and participants were approached after completing their regular medical care to ensure minimal disruption.

### Data quality assurance

The questionnaire was first prepared in English, translated into the local language (Gedioffa and Amharic) by a bilingual translator, and then back-translated into English by another bilingual translator to ensure consistency. The validation of the data collection tool for local languages was checked by incorporating two experts in the field to assess the translated questionnaire for face validity. In addition, to ensure the questionnaires clarity and ease of use, a pretest was conducted in 26 (5% of the total sample size) adult diabetes patients at DUTH. These participants were excluded from the final analysis. Based on the pretest results and experts feedback, minor modifications were made to the questionnaire to enhance clarity and consistency of wording before actual data collection began. The reliability (internal consistency) of the instruments was determined using Cronbach’s alpha coefficient and found to be 0.87 for the general diabetic distress scale,0.83 for depression, 0.79 for substance use, and 0.67 for the social support scale. Before the actual data collection, the principal investigator gave two days of training to the data collectors and supervisors about the objectives of the study, questionnaires, and data collection techniques. Each questionnaire was reviewed to ensure completeness, accuracy, and consistency of collected data.

### Data processing and analysis

After the data collection, the completeness and consistency of the questionnaires were checked manually, and before analysis, missing values were checked by the principal investigator. Then both the questionnaires and the variables are coded, categorized, and entered into Epi Data version 7.2 and exported to SPSS version 25 for cleaning and further analysis. The analysis was supported using descriptive interpretation, frequency tables, and summary statistics (mean and median) to summarize the socio-demographic, clinical, and patient characteristics of the study participants. Multi-collinearity between independent variables was checked using the variance inflation factor (VIF) and tolerance. The values of both statistics for all independent variables were VIF < 10 and tolerance > 0.1. In this study, the proportional odds assumptions were checked with the test of the parallel line, which indicates a p-value of 0.587. Ordinal logistic regression was carried out using a generalized linear model in SPSS to identify the association between each variable with the outcome variable. Variables with a p-value < 0.25 in the bivariable ordinal logistic regression became candidates for multivariable ordinal logistic regression. Then, factors that have a p-value < 0.05 were considered predictors of diabetic-related distress. The results were expressed in terms of crude and adjusted odds ratios with 95% confidence intervals. A p-value < 0.05 was used to determine statistically significant predictors of diabetes related distress.

Mediation analysis was conducted on diabetic patients to explore the causal pathway from the independent variables to the dependent variable through mediating variables. In this study, depression was identified as the mediating variable, as it was significantly associated with both the outcome variable (diabetes-related distress) and the predictor variable (social support). The analysis assessed several components of the mediation model, including the total effect of the social support on the diabetic related distress (path c), the direct effect of the social support on depression (path a), the effect of depression on diabetic related distress while controlling for the social support (path b), and the direct influence of social support on diabetic related distress after controlling for the effect of depression (path c′). The indirect impact was estimated using the product of paths a and b. This component of mediation analysis was easily tested using the PROCESS macro-model 4 in SPSS version 25. Additionally, the percentage of the effect attributed by the mediator was determined by dividing the indirect effect by the total effect (the sum of the indirect and direct effects), followed by multiplying by 100%. All results were reported in tabular form with regression coefficients, p-values, and 95% confidence intervals, based on bootstrap estimation.

### Ethical consideration

The study was carried out after a letter of approval was obtained from the ethical review committee (protocol unique number: duirb/045/23–06) of Dilla University College of Medicine and Health Science, which also facilitated an official letter to be written to the selected hospitals to get their permission and cooperation for the study. Approval was also obtained from participating hospitals. Written informed, voluntary consent was taken from each study participant who was selected for the study. Before the interview begins, the data collector assures participants that the information they give is used only for research purposes and kept anonymous throughout data collection.

## Results

### Socio-demographic characteristics of diabetic patients

A total of 506 diabetic patients fulfilled the inclusion criteria and participated. Among these, 55.1% (279) of males and 44.9% (227) of females participated in this study. The mean age of the patients was 51.12 ± 16.6 years, while the maximum and minimum ages of patients visiting the outpatient department were 88 and 18 years, respectively. In terms of marital status,54.5% (276) were married. Additionally, about 19.4%(98) of the sample had not received any formal education (Table 1).

**Table 1:** Socio-demographic characteristics of adult diabetes mellitus patients.

Variables	Categories	Frequency (%)
Sex	Male	279(55.1)
	Female	227(44.9)
Age	<52yrs	230(45.5)
	≥52yrs	276(54.5)
	Mean ± SD	51.12 ± 16.6
Marital status	Single	106(20.9)
	Married	276(54.5)
	Widow	69 (13.6)
	Divorce/Separated	55 (10.9)
Educational level	No formal education	98 (19.4)
	Able to read and write	134(26.5)
	Primary Education	125(24.7)
	Secondary Education and above	149 (29.4)
Occupation status	Unemployed	167(33.0)
	Governmental Employed	104(20.6)
	Private Employed	48(9.5)
	Farmer	68(13.4)
	Merchant	119(23.5)
Place of residence	Urban	348(68.8)
	Rural	158(31.2)

### The magnitude of diabetic-related distress among diabetic patients

**Fig 1:**
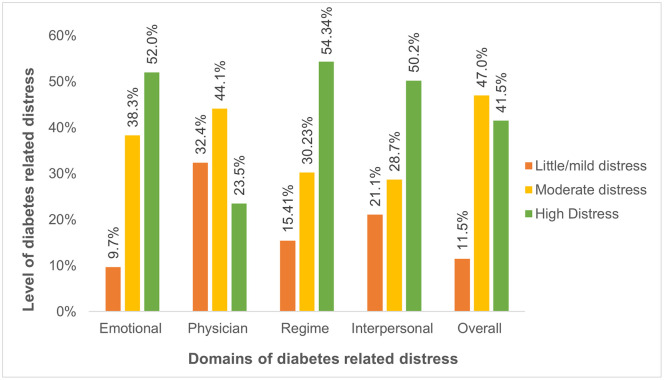
Prevalence of diabetic-related distress and its domains among adult diabetes mellitus patients.

This study found that 88.54% of diabetes patients had diabetic-related distress, in which 41.5% (95%CI; 37.2–45.9) of patients had high distress and 47.04% (95%CI;42.6–51.5) had moderate distress (Fig 1).

### Clinical-related characteristics of diabetic patients

The majority, 378(74.7%) of diabetic patients had been living with diabetes for less than 7 years, and 205(40.5%) patients had experienced diabetic-related complications; among these, 84(16.6%) developed diabetes foot ulcers. Regarding diabetic medications, 266 (52.6%) of respondents were taking both oral medication and insulin. The study also revealed that 411(81.2%) of the study participants had FBG levels of ≥ 130 mg/dl, and more than half, 379(74.9%), were diagnosed with type 2 diabetes (Table 2).

**Table 2:** Clinical-related characteristics of adult diabetes mellitus patients.

Variables	Categories	Frequency (%)
Duration of living with diabetes	≤ 7 years	378(74.7)
	8-14years	100(19.8)
	≥15years	28(5.5)
	Median [IQR]	6 [5–7]
Diabetic associated complications	Yes	205(40.5)
	No	301(59.5)
Type of complication	Nephropathy	32(6.3)
	Retinopathy	62(12.3)
	Diabetic foot ulcer	84(16.6)
	Diabetes related Coronary disease	27(5.3)
Hypoglycemic in the last 3 months	Yes	102 (20.2)
	No	404(79.8)
Type of treatment	Oral	188(37.2)
	Insulin	52 (10.3)
	Oral and insulin	266 (52.6)
Depression	Mild (4–9)	49(9.7)
	Moderate (10–19)	213(42.1)
	Sever (≥20)	244(48.2)
Fast blood glucose (FBG mg/dl)	70-99 mg/dl	39(7.7)
	100-129 mg/dl	56(11.1)
	≥130	411(81.2)
Blood pressure (mm Hg)	Normal (<120/80)	97(19.2)
	Pre-hypertension (120–139/80–89)	196(38.7)
	Hypertension (≥140/90)	213(42.1)
BMI (kg/m)	Normal weight (18.5–24.9 kg/m^2^)	16(3.2)
	Overweight (25–29.9 kg/m^2^)	158(31.2)
	Obese (≥30 kg/m^2^)	332(65.6)
Type of DM	Type 1	127(25.1)
	Type 2	379(74.9)

### Patient-related characteristics of diabetic patients

Among 506 patients attending the outpatient department, 245(39.0%) had never engaged in planned physical activity, and 446 (88.1%) had reported an average sleep duration of less than eight hours. More than half, 326(64.4%) of the patients experience poor social support (Table 3).

**Table 3:** Patient-related characteristics of adult diabetes mellitus patients.

Variables	Categories	Frequency (%)
Planned physical exercise program	Never	469(92.7)
	Once or twice a week	22(4.3)
	Regular exercise (≥ three times a week)	15(3.0)
Average Duration of Sleep (in hours)	<8hrs	446 (88.1)
	≥8hrs	60 (11.9)
	Mean ±SD	6.28 ± 1.15
Alcohol intake	Low risk	374(73.9)
	Moderate risk	95(18.8)
	High risk	37(7.3)
Cigarette smoking	Low risk	343 (67.8)
	Moderate risk	128(25.3)
	High risk	35(6.9)
Social support	Poor support	326(64.4)
	Medium support	154(30.4)
	High support	26(5.1)

### Mediation analysis

This study conducted a mediation analysis to examine whether depression serves as a mediator in the relationship between social support and diabetes-related distress among diabetic patients. The total effect of social support on diabetic distress was statistically significant (*β* = –0.0635, 95% CI [- 0.10, −0.026], *P *< 0.001), indicating that higher social support is associated with lower levels of diabetic distress. When depression was included as a mediator in the model, the direct effect of social support on diabetic distress remained statistically significant (*β* = –0.056,95% CI [- 0.0924, −0.0189], *P* = 0.003). This suggests that social support continues to have a direct impact on diabetic distress even after considering the mediating role of depression. Furthermore, the indirect effect of social support on diabetic distress through depression was also statistically significant (*β* = –0.0078, 95% CI [–0.0162, –0.0015]). This all implies that depression partially mediates and explains 12.28% of the social support effect on diabetic distress.

### Factors associated with diabetic-related distress among diabetes patients

Out of twenty-one independent variables, thirteen variables, namely age, educational status, marital status, planned exercise, treatment type, duration of diabetes, blood pressure, social support, complications, hypoglycemia, FBG, depression, and BMI, showed an association in bivariable analysis at p value < 0.25. These variables were then entered into the multivariable logistic regression analysis. Planned exercise, blood pressure status, social support, and complications were identified as statistically significant predictors of diabetic-related distress among diabetes patients with a p-value < 0.05.

Our study shows that patients who have been physically inactive or who never had planned physical activity were approximately three times more likely to have diabetic distress (aOR= 3.09;95%CI = 1.034–9.28; P = 0.04). The chance that a person who has diabetic complications was 1.79 times more likely to have diabetic-related distress than a person who did not have complications (aOR=1.79; 95%CI = 1.13–2.84; P < 0.009). In addition, patients with high blood pressure are 1.8 times more likely to experience distress than normal patients (aOR=1.85; 95%CI = 1.12–3.05; P = 0.016). Finally, patients who had lower social support were 3.4 times more likely to have diabetic-related distress than higher social support (aOR=3.44; 95%CI = 1.42–8.33; P < 0.006) (Table 4).

**Table 4:** Bivariable and multivariable ordinal logistic regression analysis for factors associated with diabetic-related distress.

Variables	Categories	DRD			OR (95%CI; P value)	aOR (95%CI)	P value
		Mild	Moderate	Severe			
Age	<52yrs	30	111	89	0.79 (0.57 - 1.1; 0.18) *	1.0(0.65-1.55)	0.98
	≥52yrs	28	127	121	Reference	Reference	
Educational status	Uneducated	16	48	34	0.72(0.44 −1.17; 0.19) *	0.63(0.37-1.07)	0.087
	Able to read and write	13	57	64	1.28(0.81-2.0; 0.28)	1.07(0.64-1.79)	0.80
	Primary Education	13	61	51	1.0(0.64-1.57; 0.99)	0.94(0.58 −1.54)	0.81
	Secondary Education and above	16	72	61	Reference	Reference	
Marital status	Single	12	49	45	0.95(0.62-1.45; 0.80)	1.31(0.76-2.28)	0.33
	Married	20	142	115	Reference	Reference	
	Widow	15	25	28	0.7(0.41-1.19; 0.23) *	0.59(0.33-1.06)	0.079
	Divorce/Separated	11	22	22	0.71(0.40-1.26; 0.18) *	0.64(0.34-1.19)	0.16
Planned Exercise	Never	49	220	200	2.5(0.87-7.21; 0.069) *	3.09(1.03-9.28) **	**0.043**
	Once or twice a week	4	13	5	1.13(0.30 - 4.17; 0.847)	1.32(0.34 −5.05)	0.68
	Regular exercise (≥ three times a week)	5	5	5	Reference	Reference	
Treatment type	Oral	25	82	81	0.93(0.65- 1.34; 0.71)	1.13 (0.75-1.7)	0.55
	Insulin	10	25	17	0.58(0.33-1.03;0.06) *	0.69 (0.35-1.36)	0.29
	Oral and insulin	23	131	112	Reference	Reference	
Duration of DM	≤ 7 years	46	185	147	Reference	Reference	
	8-14years	7	44	49	1.54(1.0-2.36; 0.04) *	1.32(0.79-2.19)	0.28
	≥15years	5	9	14	1.28(0.59 - 2.7; 0.50)	0.93(0.40-2.14)	0.87
Blood pressure status	Normal	20	47	30	Reference	Reference	
	Pre-hypertension	22	94	97	2.05(1.28-3.27; 0.006) *	1.64(0.97-2.76)	0.063
	Hypertension	16	97	83	1.93(1.20- 3.09; 0.002) *	1.85(1.12- 3.05) **	**0.016**
Social support	Poor support	10	72	72	5.08(2.19 - 11.8; < 0.001) *	3.44(1.42-8.34) **	**0.006**
	Medium support	37	157	131	3.72(1.65 −8.37; 0.001) *	2.29(0.96-5.46)	0.061
	High support	11	9	7	Reference	Reference	
Complications	Yes	12	96	97	1.67(1.19 −2.36; 0.003) *	1.79(1.13-2.84) **	**0.013**
	No	46	142	113	Reference	Reference	
Hypoglycemia	Yes	13	52	37	0.78(0.52- 1.18; 0.24) *	0.64(0.4-1.02)	0.062
	No	45	186	173	Reference	Reference	
Fast blood glucose level	70-99 mg/dl	10	15	14	Reference	Reference	
	100-129 mg/dl	8	27	21	1.41(0.63-3.16; 0.39)	0.98(0.413- 2.36)	0.97
	≥130	40	196	175	1.83(0.95-3.54; 0.06) *	1.29(0.64-2.60)	0.48
Depression	Mild (4–9)	11	19	19	Reference	Reference	
	Moderate (10–19)	33	111	69	0.97 (0.52 −1.8; 0.93)	1.66(0.86-3.187)	0.13
	Sever (≥20)	14	108	122	2.13 (1.15 −3.93; 0.01) *	0.80(0.42-1.53)	0.50
BMI	Normal weight (18.5–24.9 kg/m^2^)	34	156	142	Reference	Reference	
	Overweight (25–29.9 kg/m^2^)	19	77	62	0.86 (0.59-1.23; 0.41)	0.82(0.55 - 1.22)	0.34
	Obese (≥30 kg/m^2^)	5	5	6	0.49 (0.17-1.37, 0.14) *	0.76(0.25-2.35)	0.64

**Note**; OR= crude odd ratio; aOR= adjusted odd ratio, * = p Value <0.25; ** = p Value <0.05.

## Discussion

This study was conducted to assess the level of DRD and its associated factors among adult diabetes mellitus patients attending public hospitals in Gedio zone, southern Ethiopia. The study showed that 11.46% (95%CI;8.8–14.6) had little or mild distress, while 47.04% (95%CI;42.6–51.5) and 41.5% (95%CI; 37.2–45.9) had moderate and high levels of diabetic-related distress, respectively. The findings of this study were consistent with the study conducted in Amhara regional state [40], which reported that 43.2% of patients had a moderate level of distress and 44.4% experienced a high level of diabetic distress. Similar results were also observed in southeast Ethiopia [17], in which 41.8% of the participants suffer from a higher level of DRD. However, it is higher in comparison with previous studies conducted in Vietnam (23.6%) [41], and Barbadian(17%) [42]. The higher prevalence in this study could be due to poor quality of diabetes care service, and the measurement tool used to quantify the level of diabetic distress. The highest level of distress was observed in the Regime-related domain of diabetic-related distress, which is in line with the study done in Iran [34]. The possible explanation is that decreased medication adherence has an impact on glycemic control, which results in further complications and decreased self-management [43]. To reduce the impact of regimen-related distress resulting from poor glycemic control due to medication non-adherence, the application of cognitive-behavioral therapy may lead to significant improvements in HbA1c levels and help to improve blood glucose levels, thereby reducing distress [44]. In addition, diabetes health-coaching programs are beneficial in improving glycemic control and reducing distress [45]. The lowest level of physician-related distress in our study may be due to the patients higher satisfaction with the doctor-patient relationship [34].

Our study shows that physically inactive participants were more likely to have diabetes distress. This finding is consistence with the conclusions drawn from previous research carried out in the Amhara region and Saudi Arabia. The potential reason could be that those who did not engage in regular physical activities might think they do not sufficiently adhere to their supportive self-care management, which can lead to high regimen-related distress [40,17,46]. Patients who have complications are significantly associated with higher levels of distress, which is in line with previous studies done in southwest Ethiopia [47]. People living with diabetes may become emotionally burdened, frustrated, discouraged by the threat of developing complications, and perceive themselves as helpless to avert them. This causes distress and results in withdrawal from their self-care routines [17]. This study also found that people with high blood pressure are more likely to have DRD. The possible explanation for this finding could be the result of experienced side effects of antihypertensive medication [48]. Additionally, the odds of DRD increase in patients who had low social support, which is in line with studies done in Indonesia and southeast Ethiopia [36,17]. The possible reasons for this could be social support from family or friends, as a form of emotional, informational, or financial can help the patient cope with problems and give emotional strength. Furthermore, this study shows that a lower level of social support leads to higher diabetic related distress through the mediating variable of depression. Previous studies have separately demonstrated that poor social support is associated with increased levels of both depression [49] and diabetic related distress [50], and that depression is positively correlated with distress [51,52]. This may be explained by the fact that individuals without adequate social support may first experience feelings of loneliness, helplessness, and hopelessness. These negative behaviors increase or worsen diabetic related stressors, emotional burden, frustration, and make them more vulnerable to distress.

## Conclusion and recommendations

Generally, this study reveals that 47.1% of diabetic patients experience a moderate diabetic related distress, while 41.5% face a high level of diabetic related distress. The results further suggest that complications, high blood pressure, inadequate social support, and absence of planned physical exercise are important factors that contribute to increased levels of distress in diabetic patients. This study also indicates that depression has a significant mediation role between social support and diabetes related distress.

Healthcare facilities should implement targeted strategies to reduce diabetes-related distress by addressing the key contributing factors identified in this study. This includes integrating complication monitoring into routine follow-up visits, which enables healthcare providers to regularly screen for complications and offer timely interventions to ease both the physical and emotional burdens on patients. Additionally, giving training for social health workers to assess patients social support status and link them to counseling can strengthen family and social connections, thereby helping to reduce distress. Regarding depression, due to its significant effect on social support and diabetic distress as a mediator, healthcare providers should plan to incorporate routine screening and effective management of depression into patient care. Furthermore, addressing comorbid conditions such as hypertension and promoting emotional well-being should be integrated into routine care. Moreover, healthcare providers are encouraged to offer individualized exercise counseling to help patients establish and maintain a regular physical activity routine. Finally, we recommend that future researchers should explore longitudinal and intervention-based designs to better understand the causal pathway and to test the effectiveness of a specific intervention in reducing DRD.

### Limitations of the study

Despite this study providing meaningful contributions, it has some limitations that should be considered when interpreting the results. First, the cross-sectional nature of the study only determines the association between variables, not the causality or direction. Therefore, future experimental studies are required to identify the causality between variables. Second, the data collected is based on self-reporting and might be subject to recall and response bias, which might influence the result. Participants in this study were selected from hospital patients, which may introduce selection bias because people in the hospital may have severe DM, and individuals who can manage their condition well at home are often missing from the sample. Thirdly, it is difficult to determine whether the distress occurred before the onset of diabetes or is entirely associated with the disease. Finally, some participants stated that they were in a hurry to get to work and did not have time due to their busy schedules. As a result, they were unwilling to complete the questionnaire and were recorded as non-respondents. In the future, if a participant is unable to make time for an in-person interview, it is better to ask the participant to complete the questionnaire through a phone call or email.

## Supporting information

S1 Questionnaires English version questionnaire for diabetic-related distress and associated factors among adult diabetes mellitus patients attending public hospitals in Gedio zone, southern Ethiopia. (DOCX)

S2 Questionnaires Amharic version questionnaire for diabetic-related distress and associated factors among adult diabetes mellitus patients attending public hospitals in Gedio zone, southern Ethiopia. (DOCX)

S3 Questionnaires Gedioffa version questionnaire for diabetic-related distress and associated factors among adult diabetes mellitus patients attending public hospitals in Gedio zone, southern Ethiopia. (DOCX)

S4 Data Overall data on diabetic-related distress and associated factors among adult diabetes mellitus patients attending public hospitals in Gedio zone, southern Ethiopia. (XLSX)

## References

[1] American Diabetes Association. Introduction: standards of medical care in diabetes-2022. Diabetes Care. 2022;45(Suppl 1):S1–2. doi: 10.2337/dc22-Sint 34964812

[2] Roglic G. WHO global report on diabetes: A summary. Int J Noncommun Dis. 2016;1(1):3–8.

[3] Kovács N, Shahin B, Andrade CAS, Mahrouseh N, Varga O. Lifestyle and metabolic risk factors, and diabetes mellitus prevalence in European countries from three waves of the European Health Interview Survey. Sci Rep. 2024;14(1):11623. doi: 10.1038/s41598-024-62122-y 38773149 PMC11109107

[4] Toi PL, Anothaisintawee T, Chaikledkaew U, Briones JR, Reutrakul S, Thakkinstian A. Preventive role of diet interventions and dietary factors in type 2 diabetes mellitus: an umbrella review. Nutrients. 2020;12(9):2722. doi: 10.3390/nu12092722 32899917 PMC7551929

[5] Hendrieckx C. Diabetes and emotional health: a practical guide for health professionals supporting adults with type 1 or type 2 diabetes. Deakin University; 2020. 10.2196/15007 PMC7060499 32130112

[6] St Quinton T. Applying the reasoned action approach and planning to understand diabetes self-management behaviors. Behav Sci (Basel). 2022;12(10):375. doi: 10.3390/bs12100375 36285944 PMC9598101

[7] American Diabetes Association. Standards of Medical Care in Diabetes-2022 Abridged for primary care providers. Clin Diabetes. 2022;40(1):10–38. doi: 10.2337/cd22-as01 35221470 PMC8865785

[8] Stanković Z, Jasović-Gasić M, Lecić-Tosevski D. Psychological problems in patients with type 2 diabetes--clinical considerations. Vojnosanit Pregl. 2013;70(12):1138–44. doi: 10.2298/vsp1312138s 24450259

[9] Polonsky WH, Fisher L, Earles J, Dudl RJ, Lees J, Mullan J, et al. Assessing psychosocial distress in diabetes: development of the diabetes distress scale. Diabetes Care. 2005;28(3):626–31. doi: 10.2337/diacare.28.3.626 15735199

[10] Sendekie AK, Limenh LW, Bizuneh GK, Kasahun AE, Wondm SA, Tamene FB, et al. Psychological distress and its impact on glycemic control in patients with diabetes, Northwest Ethiopia. Front Med (Lausanne). 2025;12:1488023. doi: 10.3389/fmed.2025.1488023 40206466 PMC11979121

[11] Xing S, Liu Y, Zhang H, Li B, Jiang X. The mediating role of diabetes stigma and self-efficacy in relieving diabetes distress among patients with type 2 diabetes mellitus: a multicenter cross-sectional study. Front Psychol. 2023;14:1147101. doi: 10.3389/fpsyg.2023.1147101 37575426 PMC10416640

[12] Guo X, Wong PN, Koh YL, Tan NC. Factors associated with diabetes-related distress among Asian patients with poorly controlled type-2 diabetes mellitus: a cross-sectional study in primary care. 2022. 10.1186/s12875-023-02012-w PMC9969642 36849921

[13] Hayashino Y, Okamura S, Tsujii S, Ishii H, Diabetes Distress and Care Registry at Tenri Study Group. Association between diabetes distress and all-cause mortality in Japanese individuals with type 2 diabetes: a prospective cohort study (Diabetes Distress and Care Registry in Tenri [DDCRT 18]). Diabetologia. 2018;61(9):1978–84. doi: 10.1007/s00125-018-4657-4 29947921

[14] Yachmaneni A Jr, Jajoo S, Mahakalkar C, Kshirsagar S, Dhole S. A Comprehensive review of the vascular consequences of diabetes in the lower extremities: current approaches to management and evaluation of clinical outcomes. Cureus. 2023;15(10):e47525. doi: 10.7759/cureus.47525 38022307 PMC10664734

[15] Poonoosamy J, Lopes P, Huret P, Dardari R, Penfornis A, Thomas C, et al. Impact of intensive glycemic treatment on diabetes complications-a systematic review. Pharmaceutics. 2023;15(7):1791. doi: 10.3390/pharmaceutics15071791 37513978 PMC10383300

[16] Ndumele CE, Rangaswami J, Chow SL, Neeland IJ, Tuttle KR, Khan SS, et al. Cardiovascular-kidney-metabolic health: a presidential advisory from the American Heart Association. Circulation. 2023;148(20):1606–35. doi: 10.1161/CIR.0000000000001184 37807924

[17] Adugnew M, Fetene D, Assefa T, Kedir S, Asmamaw K, Feleke Z, et al. Diabetes-related distress and its associated factors among people with type 2 diabetes in Southeast Ethiopia: a cross-sectional study. BMJ Open. 2024;14(1):e077693. doi: 10.1136/bmjopen-2023-077693 38176868 PMC10773350

[18] Jemal M, Argaw A, Taye A, Sintayehu T, Kedir S. Dietary self-care and associated factors among diabetic patients in Jimma University Medical Centre, South West Ethiopia; a path analysis. PLoS One. 2022;17(8):e0273074. doi: 10.1371/journal.pone.0273074 36001578 PMC9401131

[19] Tanaka N, Yabe D, Murotani K, Ueno S, Kuwata H, Hamamoto Y, et al. Mental distress and health-related quality of life among type 1 and type 2 diabetes patients using self-monitoring of blood glucose: A cross-sectional questionnaire study in Japan. J Diabetes Investig. 2018;9(5):1203–11. doi: 10.1111/jdi.12827 29493881 PMC6123045

[20] Downie GA, Mullan BA, Boyes ME, McEvoy PM. The effect of psychological distress on self-care intention and behaviour in young adults with type 1 diabetes. J Health Psychol. 2021;26(4):543–55. doi: 10.1177/1359105318824795 30666886

[21] Elotla SF, Fouad AM, Mohamed SF, Joudeh AI, Mostafa M, Hayek SE, et al. Association between diabetes-related distress and glycemic control in primary care patients with Type 2 diabetes during the coronavirus disease 2019 (COVID-19) pandemic in Egypt. J Family Community Med. 2023;30(1):42–50. doi: 10.4103/jfcm.jfcm_238_22 36843865 PMC9954422

[22] Al Sayah F, Yeung RO, Johnson JA. Association of depressive symptoms and diabetes distress with severe hypoglycemia in adults with type 2 diabetes. Can J Diabetes. 2019;43(5):316–21. doi: 10.1016/j.jcjd.2018.11.002 30578165

[23] Carper MM, Traeger L, Gonzalez JS, Wexler DJ, Psaros C, Safren SA. The differential associations of depression and diabetes distress with quality of life domains in type 2 diabetes. J Behav Med. 2014;37(3):501–10. doi: 10.1007/s10865-013-9505-x 23515932 PMC3758402

[24] Stephani V, Opoku D, Beran D. Self-management of diabetes in Sub-Saharan Africa: a systematic review. BMC Public Health. 2018;18(1):1148. doi: 10.1186/s12889-018-6050-0 30268115 PMC6162903

[25] Geleta BA, Dingata ST, Emanu MD, Eba LB, Abera KB, Tsegaye D. Prevalence of diabetes related distress and associated factors among type 2 diabetes patients attending hospitals, Southwest Ethiopia, 2020: a cross-sectional study. Patient Relat Outcome Meas. 2021;12:13–22. doi: 10.2147/PROM.S290412 33542669 PMC7850978

[26] Population EO, H C C. Summary and statistical report of the 2007 population and housing census: Population size by age and sex. Federal Democratic Republic of Ethiopia, Population Census Commission; 2008.

[27] Nguyen VB, Tran TT, Dang TL, Nguyen VVH, Tran BT, Le CV, et al. Diabetes-related distress and its associated factors among patients with diabetes in Vietnam. Psychol Res Behav Manag. 2020;13:1181–9. doi: 10.2147/PRBM.S285291 33363418 PMC7754255

[28] Fisher L, et al. When is diabetes distress clinically meaningful? Establishing cut points for the Diabetes Distress Scale. Diabetes Care. 2012;35(2):259–64. 22228744 10.2337/dc11-1572 PMC3263871

[29] American Diabetes Association. Diagnosis and classification of diabetes mellitus. Diabetes Care. 2014;37 Suppl 1:S81–90. doi: 10.2337/dc14-S081 24357215

[30] Abiola T, Udofia O, Zakari M. Psychometric properties of the 3-item oslo social support scale among clinical students of Bayero University Kano, Nigeria. Malaysian J Psychiatry. 2013;22(2):32–41.

[31] Lasebikan VO, Ola BA. Prevalence and correlates of alcohol use among a sample of Nigerian semirural community dwellers in Nigeria. J Addict. 2016;2016:2831594. doi: 10.1155/2016/2831594 27195170 PMC4853965

[32] Lasebikan V, Ola BA, Ayinde OO. Effectiveness of alcohol, smoking, and substance involvement screening test-linked brief intervention on harmful and hazardous alcohol use in Nigerian Semirural Communities: a non-randomized intervention study. Front Psychiatry. 2017;8:50. doi: 10.3389/fpsyt.2017.00050 28443034 PMC5385697

[33] Ismail M, Seif MH, Metwally N, Neshnash M, Joudeh AI, Alsaadi M, et al. Prevalence and determinants of depression among patients with Type 2 diabetes mellitus attending family medicine clinics in Qatar. Am J Med Open. 2022;9:100014. doi: 10.1016/j.ajmo.2022.100014 39035064 PMC11256244

[34] Khashayar P, Shirzad N, Zarbini A, Esteghamati A, Hemmatabadi M, Sharafi E. Diabetes-related distress and its association with the complications of diabetes in Iran. J Diabetes Metab Disord. 2022;21(2):1569–75. doi: 10.1007/s40200-022-01103-2 35915591 PMC9328774

[35] Ramkisson S, Pillay BJ, Sartorius B. Diabetes distress and related factors in South African adults with type 2 diabetes. J Endocrinol Metabolism Diabetes South Africa. 2016;21(2):35–9.

[36] Bhaskara G, Budhiarta AAG, Gotera W, Saraswati MR, Dwipayana IMP, Semadi IMS, et al. Factors Associated with diabetes-related distress in type 2 diabetes mellitus patients. Diabetes Metab Syndr Obes. 2022;15:2077–85. doi: 10.2147/DMSO.S363431 35873530 PMC9296679

[37] Fukuda N, Gandhi K, Lim E, Leake A. Validation of the diabetes distress scale in an Asian Pacific Islander population. Hawaii J Med Public Health. 2019;78(1):3–7. 30697468 PMC6333958

[38] M. A, Khapre M, Kant R, Kumar P. Diabetes-related distress: translation and validation of the Hindi version of Diabetes Distress Scale (DDS) for Indian type 2 diabetes mellitus patients. Int J Diabetes Dev Ctries. 2024;45(1):141–9. doi: 10.1007/s13410-024-01338-0

[39] Chew B, et al. The reliability and validity of the Malay version 17-item diabetes distress scale. Malaysian Family Phys. 2015;10(2):22. PMC4826578 27099658

[40] Wolde AK. Diabetic distress among diabetic patients in the Amhara Regional State, Ethiopia. Commun Health Equity Res Policy. 2023;43(2):171–81. doi: 10.1177/0272684X211004931 33823688

[41] Huynh G, Tran TT, Do THT, Truong TTD, Ong PT, Nguyen TNH, et al. Diabetes-related distress among people with type 2 diabetes in Ho Chi Minh City, Vietnam: prevalence and associated factors. Diabetes Metab Syndr Obes. 2021;14:683–90. doi: 10.2147/DMSO.S297315 33623403 PMC7894807

[42] DaSantos A, Goddard C, Ragoobirsingh D. Diabetes distress in Barbadian adults with type 2 diabetes. AIMS Public Health. 2022;9(3):471–81. doi: 10.3934/publichealth.2022032 36330278 PMC9581746

[43] Hessler D, Fisher L, Glasgow RE, Strycker LA, Dickinson LM, Arean PA, et al. Reductions in regimen distress are associated with improved management and glycemic control over time. Diabetes Care. 2014;37(3):617–24. doi: 10.2337/dc13-0762 24170750 PMC3931383

[44] Tunsuchart K, Lerttrakarnnon P, Srithanaviboonchai K, Likhitsathian S, Skulphan S. Benefits of brief group cognitive behavioral therapy in reducing diabetes-related distress and HbA1c in uncontrolled type 2 diabetes mellitus Patients in Thailand. Int J Environ Res Public Health. 2020;17(15):5564. doi: 10.3390/ijerph17155564 32752228 PMC7432874

[45] Sherifali D, Brozic A, Agema P, Punthakee Z, McInnes N, O’Reilly D, et al. Effect of diabetes health coaching on glycemic control and quality of life in adults living with type 2 diabetes: a community-based, randomized, controlled trial. Can J Diabetes. 2021;45(7):594–600. doi: 10.1016/j.jcjd.2020.11.012 33582039

[46] Alzughbi T, Badedi M, Darraj H, Hummadi A, Jaddoh S, Solan Y, et al. Diabetes-related distress and depression in saudis with type 2 diabetes. Psychol Res Behav Manag. 2020;13:453–8. doi: 10.2147/PRBM.S255631 32547267 PMC7239888

[47] Geleta BA, et al. Prevalence of diabetes related distress and associated factors among type 2 diabetes patients attending hospitals, Southwest Ethiopia, 2020: a cross-sectional study. Patient Related Outcome Meas. 2021:13–22. 10.2147/PROM.S290412 PMC7850978 33542669

[48] Chew B-H, Vos R, Mohd-Sidik S, Rutten GEHM. Diabetes-related distress, depression and distress-depression among adults with type 2 diabetes mellitus in Malaysia. PLoS One. 2016;11(3):e0152095. doi: 10.1371/journal.pone.0152095 27002728 PMC4803274

[49] Diress G, Endalifer ML, Addisu A, Mengist B. Association between social supports and depression among patients with diabetes mellitus in Ethiopia: a systematic review and meta-analysis. BMJ Open. 2022;12(5):e061801. doi: 10.1136/bmjopen-2022-061801 35545384 PMC9096548

[50] Presley CA, Mondesir FL, Juarez LD, Agne AA, Riggs KR, Li Y, et al. Social support and diabetes distress among adults with type 2 diabetes covered by Alabama Medicaid. Diabet Med. 2021;38(4):e14503. doi: 10.1111/dme.14503 33351189 PMC7979501

[51] Kamrul-Hasan ABM, Hannan MA, Asaduzzaman M, Rahman MM, Alam MS, Amin MN, et al. Prevalence and predictors of diabetes distress among adults with type 2 diabetes mellitus: a facility-based cross-sectional study of Bangladesh. BMC Endocr Disord. 2022;22(1):28. doi: 10.1186/s12902-022-00938-3 35065623 PMC8783990

[52] Riise HKR, Haugstvedt A, Igland J, Graue M, Søfteland E, Hermann M, et al. Diabetes distress and associated psychosocial factors in type 2 diabetes. A population-based cross-sectional study. The HUNT study, Norway. Diabetol Metab Syndr. 2025;17(1):62. doi: 10.1186/s13098-025-01631-w 39972441 PMC11837721

