# Supplementary material for: Diabetes-related distress and associated factors among adult diabetes mellitus patients attending public hospitals in Gedio zone, southern Ethiopia: Mediation analysis
Source: PLoS One. 2025 Sep 29;20(9):e0331655. doi: 10.1371/journal.pone.0331655 (PMC12478905; doi:10.1371/journal.pone.0331655)
Supplement: S1 Questionnaires — (DOCX) [file pone.0331655.s001.docx]

**English Version Questioner**

**Participants Information sheet**

Dear Participants

Hello! My name is ………………...... I am here on behalf of one of the researchers at Dilla University, College of Medicine and Health Science. They are undertake research on diabetic-related distress among diabetic Mellitus patients and received permission from the university and the respective hospitals. This study will help identify factors related to diabetic-related distress. It will also serve as a baseline for other wide studies as well as for planning health intervention activities in the country. The data collected from you is very important for this study. Therefore, your help in providing the data in a comfortable manner is crucial for this study. Your participation is completely voluntary. You can refuse to answer any questions and/or withdraw from the study at any time without any problem to you or the services you get in the hospital. All your responses will remain strictly confidential: the hospital staff will not have access to your responses; your name will not appear on the interview and will not be recorded. If there are any questions or inquiries at any time about the study, you can contact the principal investigator via data collector personnel at any point in time.

Based on your understanding of the information, are you willing to participate in the Study?

Yes__________ (continue) No_______________ Thank you!

**Respondent**

Signature ______________________

**Instruction:** Data will be collected from each diabetic mellitus patient (April to May, 2024). Please give an appropriate response for each question.

| **part I: Variables related to socio-demographic characteristics** | | | | | | | | | | | | | | | | |
| --- | --- | --- | --- | --- | --- | --- | --- | --- | --- | --- | --- | --- | --- | --- | --- | --- |
| SNO | Questions | | | Answer | | | | | | Codes | | | Skipping | | | Remarks |
| 101 | Sex | | | Male | | | | | | 1 | | |  | | |  |
|  |  |  |  | Female | | | | | | 2 | | |  |  |  |  |
| 102 | Age | | | ------------------ | | | | | |  | | |  | | |  |
| 103 | Marital status | | | Single | | | | | | 1 | | |  | | |  |
|  |  |  |  | Married | | | | | | 2 | | |  |  |  |  |
|  |  |  |  | Widow | | | | | | 3 | | |  |  |  |  |
|  |  |  |  | Divorce /Separated | | | | | | 4 | | |  |  |  |  |
| 104 | Educational level | | | No formal education | | | | | | 1 | | |  | | |  |
|  |  |  |  | Able to read and write | | | | | | 2 | | |  |  |  |  |
|  |  |  |  | Primary Education | | | | | | 3 | | |  |  |  |  |
|  |  |  |  | Secondary Education and above | | | | | | 4 | | |  |  |  |  |
| 105 | Occupation status | | | Unemployed | | | | | | 1 | | |  | | |  |
|  |  |  |  | Governmental Employed | | | | | | 2 | | |  |  |  |  |
|  |  |  |  | Private Employed | | | | | | 3 | | |  |  |  |  |
|  |  |  |  | Farmer | | | | | | 4 | | |  |  |  |  |
|  |  |  |  | Merchant | | | | | | 5 | | |  |  |  |  |
|  |  |  |  | Others …………………….. | | | | | |  | | |  |  |  |  |
| 106 | Place of residence | | | Urban | | | | | | 1 | | |  | | |  |
|  |  |  |  | Rural | | | | | | 2 | | |  |  |  |  |
| **Part II: Diabetic Distress Scale (DDS-17) Questionnaires** | | | | | | | | | | | | | | | | |
| Each of the items may have distressed or bothered you during the past month and put a check **(√)** for the appropriate number | | | | | | | | | | | | | | | | |
| Questioners | | Not a problem (1) | | | Slight problem (2) | Moderate problem (3) | | Somewhat problem (4) | | | | Serious problem (5) | | Very serious problem  (6) | | |
| 201 | Feeling that diabetes is taking up too much of my mental and physical energy every day |  | | |  |  | |  | | | |  | |  | | |
| 202 | Feeling that my doctor doesn’t know enough about diabetes and diabetes care |  | | |  |  | |  | | | |  | |  | | |
| 203 | Feeling angry, scared, and /or depressed when I think about living with diabetes |  | | |  |  | |  | | | |  | |  | | |
| 204 | Feeling that my doctor doesn’t give me enough directions on how to manage my diabetes |  | | |  |  | |  | | | |  | |  | | |
| 205 | Feeling that I am not testing my blood sugar frequently enough |  | | |  |  | |  | | | |  | |  | | |
| 206 | Feeling that I am often failing with my diabetes routine |  | | |  |  | |  | | | |  | |  | | |
| 207 | Feeling that friends or family are not supportive enough of my self-care effort (e.g., planning activities that conflict with my schedule, encouraging me to eat the “wrong” food |  | | |  |  | |  | | | |  | |  | | |
| 208 | Feeling that diabetes controls my life |  | | |  |  | |  | | | |  | |  | | |
| 209 | Feeling that my doctor doesn’t take my concerns seriously enough |  | | |  |  | |  | | | |  | |  | | |
| 210 | Not feeling confident in my day to day ability to manage diabetes |  | | |  |  | |  | | | |  | |  | | |
| 211 | Feeling that I will end up with serious long-term complications, no matter what I do |  | | |  |  | |  | | | |  | |  | | |
| 212 | Feeling that I am not sticking closely enough to a good meal plan |  | | |  |  | |  | | | |  | |  | | |
| 213 | Feeling that friends or family doesn’t appreciate how difficult living with diabetes can be |  | | |  |  | |  | | | |  | |  | | |
| 214 | Feeling Overwhelmed by the demands of living with diabetes |  | | |  |  | |  | | | |  | |  | | |
| 215 | Feeling that I don’t have a doctor who I can see regularly enough about my diabetes |  | | |  |  | |  | | | |  | |  | | |
| 216 | Not feeling motivated to keep up my diabetes self-management |  | | |  |  | |  | | | |  | |  | | |
| 217 | Feeling that friends or family don’t give me the emotional support that I would like |  | | |  |  | |  | | | |  | |  | | |
| **Part IIIA: Variables related to** **Clinical related characteristics of patients** | | | | | | | | | | | | | | | | |
| 301 | How long have you been living with diabetes? | | | ------------------------ | | | | | |  | | |  | | |  |
| 302 | Do you have a Diabetic Associate complication | | | Yes | | | | | | 1 | | |  | | |  |
|  |  |  |  | No | | | | | | 2 | | |  |  |  |  |
| 303 | If “yes,” which type of complication does the patient have | | | Nephropathy | | | | | | 1 | | |  | | |  |
|  |  |  |  | Retinopathy | | | | | | 2 | | |  |  |  |  |
|  |  |  |  | Diabetic foot ulcer | | | | | | 3 | | |  |  |  |  |
|  |  |  |  | Others…….. | | | | | |  | | |  |  |  |  |
| 304 | Do you experienced Hypoglycemia events in the last 3 months | | | Yes | | | | | | 1 | | |  | | |  |
|  |  |  |  | No | | | | | | 2 | | |  |  |  |  |
| 305 | What type of treatment/Management do you take currently ***(Possible to select more than one alternative)*** | | | Oral | | | | | | 1 | | |  | | |  |
|  |  |  |  | Insulin | | | | | | 2 | | |  |  |  |  |
|  |  |  |  | Oral and insulin | | | | | | 3 | | |  |  |  |  |
| **Assessment of Depression Using Patient Health Questioner PHQ-9** | | | | | | | | | | | | | | | | |
|  | Over the last 2 weeks, how often have you been bothered by any of the following problems (use “√” to indicate your answer) | | | | | | | | | | | | | | | |
| 306 | Questions | | | Not at all  (0) | | | Several days (1) | | More than half the days (2) | | | | | | every day (3) | |
| 307 | Little interest or pleasure in doing things | | |  | | |  | |  | | | | | |  | |
| 308 | Feeling down, depressed, or hopeless | | |  | | |  | |  | | | | | |  | |
| 309 | Trouble falling or staying asleep, or sleeping too much | | |  | | |  | |  | | | | | |  | |
| 310 | Feeling tired or having little energy | | |  | | |  | |  | | | | | |  | |
| 311 | Poor appetite or overeating | | |  | | |  | |  | | | | | |  | |
| 312 | Feeling bad about yourself or that you are a failure or have let yourself or your family down | | |  | | |  | |  | | | | | |  | |
| 313 | Trouble concentrating on things, such as reading the newspaper or watching television | | |  | | |  | |  | | | | | |  | |
| 314 | Moving or speaking so slowly that other people could have noticed. Or the opposite being so fidgety or restless that you have been moving around a lot more than usual | | |  | | |  | |  | | | | | |  | |
| 315 | Thoughts that you would be better off dead, or of hurting yourself | | |  | | |  | |  | | | | | |  | |
| **Part IIIB: Clinical relate characteristics questioners to be filled by reviewing patients’ chart** | | | | | | | | | | | | | | | | |
| 316 | Current Level of Fast Blood Glucose (mg/dl) | | …………………… | | | | | | |  | | |  | | |  |
| 317 | Systolic blood pressure level of the patient (mmHg) | | ------------------------ | | | | | | |  | | |  | | |  |
| 318 | Diastolic blood pressure of the patient(mmHg) | | ……………………. | | | | | | |  | | |  | | |  |
| 319 | Height of the patient (in meters) | | ……………………. | | | | | | |  | | |  | | |  |
| 320 | Weight of the patient (in Kg) | | …………………….. | | | | | | |  | | |  | | |  |
| 321 | Type of DM | | Type 1 | | | | | | | 1 | | |  | | |  |
|  |  |  | Type 2 | | | | | | | 2 | | |  |  |  |  |
| **Part IV: Variables related to patient characteristics** | | | | | | | | | | | | | | | | |
| 401 | Do you have a planned physical exercise program | | | Never | | | | | | | 1 | |  | | |  |
|  |  |  |  | Once/Twice a week | | | | | | | 2 | |  |  |  |  |
|  |  |  |  | Regular exercise (>three times) | | | | | | | 3 | |  |  |  |  |
| 402 | Average Duration of Sleep | | | ………………………… | | | | | | |  | |  | | |  |
| **ASSIST Questionnaire to Assess Substance Use** | | | | | | | | | | | | | | | | |
| In your life, which of the following substances have you ever used? | | | | | | | | | | | | | | | | |
| 403 | Tobacco products (cigarettes, chewing tobacco, cigars, etc) | | | Yes | | | | | | | 1 | |  | | |  |
|  |  |  |  | No | | | | | | | 2 | |  |  |  |  |
| 404 | Alcoholic beverages (beer, wine etc) | | | Yes | | | | | | | 1 | |  | | |  |
|  |  |  |  | No | | | | | | | 2 | |  |  |  |  |
| 405 | Cannabis (marijuana, grass, hash) | | | Yes | | | | | | | 1 | |  | | |  |
|  |  |  |  | No | | | | | | | 2 | |  |  |  |  |
| 406 | Cocaine (coke) | | | Yes | | | | | | | 1 | |  | | |  |
|  |  |  |  | No | | | | | | | 2 | |  |  |  |  |
| 407 | Amphetamine-type stimulants | | | Yes | | | | | | | 1 | |  | | |  |
|  |  |  |  | No | | | | | | | 2 | |  |  |  |  |
| 408 | Inhalants (nitrous, petrol, paint thinner, etc) | | | Yes | | | | | | | 1 | |  | | |  |
|  |  |  |  | No | | | | | | | 2 | |  |  |  |  |
| 409 | Sedatives or sleeping pill (diazepam, alprazolam, flunitiazepam, midazolam, etc ) | | | Yes | | | | | | | 1 | |  | | |  |
|  |  |  |  | No | | | | | | | 2 | |  |  |  |  |
| 410 | Hallucinogens (mushrooms, ketamine, etc) | | | Yes | | | | | | | 1 | |  | | |  |
|  |  |  |  | No | | | | | | | 2 | |  |  |  |  |
| 411 | Opioids (heroin, morphine, methadone, buprenorphine, etc) | | | Yes | | | | | | | 1 | |  | | |  |
|  |  |  |  | No | | | | | | | 2 | |  |  |  |  |
| 412 | Other-specify | | | …………………. | | | | | | |  | |  | | |  |
| 413 | If “yes” to any of the above items, in the past three months how often have you used the substances you mentioned? | | | Never | | | | | | | 0 | |  | | |  |
|  |  |  |  | Once or twice | | | | | | | 2 | |  |  |  |  |
|  |  |  |  | Monthly (1 to 3 times per month) | | | | | | | 3 | |  |  |  |  |
|  |  |  |  | Weekly (1 to 4 times per week) | | | | | | | 4 | |  |  |  |  |
|  |  |  |  | Daily (5 to 7 times per week) | | | | | | | 6 | |  |  |  |  |
| 414 | If “yes” to any of the above items, during the past three months how often have you had strong desire or urge to use | | | Never | | | | | | | 0 | |  | | |  |
|  |  |  |  | Once or twice | | | | | | | 3 | |  |  |  |  |
|  |  |  |  | Monthly (1 to 3 times per month) | | | | | | | 4 | |  |  |  |  |
|  |  |  |  | Weekly (1 to 4 times per week) | | | | | | | 5 | |  |  |  |  |
|  |  |  |  | Daily (5 to 7 times per week) | | | | | | | 6 | |  |  |  |  |
| 415 | If “yes” to any of the above items, during the past three months, how often has your use of drug led to health, social, legal or financial problem | | | Never | | | | | | | 0 | |  | | |  |
|  |  |  |  | Once or twice | | | | | | | 4 | |  |  |  |  |
|  |  |  |  | Monthly (1 to 3 times per month) | | | | | | | 5 | |  |  |  |  |
|  |  |  |  | Weekly (1 to 4 times per week) | | | | | | | 6 | |  |  |  |  |
|  |  |  |  | Daily (5 to 7 times per week) | | | | | | | 7 | |  |  |  |  |
| 416 | If “yes” to any of the above items, during the past three months, how often have you failed to do what was normally expected of you because of your use of drug | | | Never | | | | | | | 0 | |  | | |  |
|  |  |  |  | Once or twice | | | | | | | 5 | |  |  |  |  |
|  |  |  |  | Monthly (1 to 3 times per month) | | | | | | | 6 | |  |  |  |  |
|  |  |  |  | Weekly (1 to 4 times per week) | | | | | | | 7 | |  |  |  |  |
|  |  |  |  | Daily (5 to 7 times per week) | | | | | | | 8 | |  |  |  |  |
| 417 | If “yes” to any of the above items, has a friend or relative or anyone else ever expressed concern about your use of drug | | | Never | | | | | | | 0 | |  | | |  |
|  |  |  |  | Yes “in the past 3 months” | | | | | | | 6 | |  |  |  |  |
|  |  |  |  | Yes, but not in the past 3 months | | | | | | | 3 | |  |  |  |  |
| 418 | If “yes” to any of the above items, have you ever tried to cut down on using drug but failed | | | Never | | | | | | | 0 | |  | | |  |
|  |  |  |  | Yes “in the past 3 months” | | | | | | | 6 | |  |  |  |  |
|  |  |  |  | Yes, but not in the past 3 months | | | | | | | 3 | |  |  |  |  |
| **Oslo Social Support Scale Questioners** | | | | | | | | | | | | | | | | |
| 414 | How many people are so close to you that you can count on them if you have a great personal problem | | | None | | | | | | | 1 | |  | | |  |
|  |  |  |  | “1-2” | | | | | | | 2 | |  |  |  |  |
|  |  |  |  | “3-5” | | | | | | | 3 | |  |  |  |  |
|  |  |  |  | 5^+^ | | | | | | | 4 | |  |  |  |  |
| 415 | How much interest and concern do people show in what you do | | | None | | | | | | | 1 | |  |  |  |  |
|  |  |  |  | Little | | | | | | | 2 | |  |  |  |  |
|  |  |  |  | Uncertain | | | | | | | 3 | |  |  |  |  |
|  |  |  |  | Some | | | | | | | 4 | |  |  |  |  |
|  |  |  |  | A lot | | | | | | | 5 | |  |  |  |  |
| 416 | How easy is it to get practical help from a neighbor if you should need it | | | Very difficult | | | | | | | 1 | |  |  |  |  |
|  |  |  |  | Difficult | | | | | | | 2 | |  |  |  |  |
|  |  |  |  | Possible | | | | | | | 3 | |  |  |  |  |
|  |  |  |  | Easy | | | | | | | 4 | |  |  |  |  |
|  |  |  |  | Very easy | | | | | | | 5 | |  |  |  |  |

**THANK YOU!**
