# Supplementary material for: Diabetes-related distress and associated factors among adult diabetes mellitus patients attending public hospitals in Gedio zone, southern Ethiopia: Mediation analysis
Source: PLoS One. 2025 Sep 29;20(9):e0331655. doi: 10.1371/journal.pone.0331655 (PMC12478905; doi:10.1371/journal.pone.0331655)
Supplement: S2 Questionnaires — (DOCX) [file pone.0331655.s002.docx]

**አማረኛ መጠየቂያ ቅጽ**

**የተሳታፊዎች መረጃ ወረቀት**

ውድ ተሳታፊዎች

ጤና ይስጥልኝ እኔ ................................................. እባላለሁ፡፡ በዲላ ዩንቨርስቲ ፤ ሕክምና እና ጤና ሳይንስ ኮሌጅ ውስጥ የምርምር ጥናት እያካሄዱ ያሉ ተመራማሪዎችን ወክየ እዚህ እገኛለሁ፡፡ የምርምር ጥናቱ ትኩረት የሚያደርገው በስኳር ህመምተኞች ላይ ከስኳር በሽታ ጋር በተያያዙ ችግሮች ላይ ሲሆን ከ ዩኒቨርስቲው እና ከሚመለከታቸው ሆስፒታሎች ፈቃድ አግኝተዋል ። ይህ ጥናት ከስኳር ህመም ጋር የተያያዙ ችግሮችን ለመለየት ይረዳል፣ለሌሎች ሰፊ ጥናቶች እና በሀገሪቱ ውስጥ የጤና ጣልቃገብነት ተግባራትን ለማቀድ እንደ መነሻ ሆኖ ያገለግላል።ስለዚህ ጥናቱ የተሟላ እንዲሆን የእርስዎ ድጋፍ እና ትብብር በጣም አስፈላጊ ነው፡፡ በዚህ ጥናት ውስጥ የርስዎ ተሳታፊነት ሙሉ በሙሉ በርስዎ ፈቃደኝነት ላይ የተመሰረተ ነዉ፤ በዚህ ጥናት ውስጥ መሳተፍዎም ሆነ ላለመሳተፍ መወሰንዎ በሆስፒታሉ ውስጥ በሚያገኙት አገልግሎት ላይ ምንም አይነት ተጽእኖ የማይኖረው ሲሆን ቃለመጠይቁን በማንኛውም ሰአት ማቋረጥ ወይም ጥያቄዎችን አለመመለስ ይችላሉ፡፡ በጥናቱ ውስጥ ለተነሱት ጥያቄዎች የሚሰጧቸው መልሶች ሙሉ በሙሉ በምስጢር የሚጠበቁ ሲሆን የርስዎም ስም በማንኛዉም መልኩ በጥናቱ ውስጥ አይገለጽም፤ እንዲሁም የሚሰጡት ምላሽ ከርስዎ ማንነት ጋር በማንኛውም መልኩ አይያያዝም፡፡ ስለ ጥናቱ በማንኛውም ጊዜ ጥያቄዎች ካለዎት በመረጃ ስብሳቢው በኩል ዋና ተመራማሪውን ማግኘት ይችላሉ::

በመረጃው ላይ ባለዎት ግንዛቤ፣ በጥናቱ ለመሳተፍ ፈቃደኛ ነዎት?

አዎ ----------------------- (ቀጥል)

አይደለሁም ----------------------- አመሰግናለሁ!

**ምላሽ ሰጪ** **ፊርማ** ______________________

**መመሪያ** ፡ መረጃው ከሚያዚያ 2024 እስከ ግንቦት 2024 ከእያንዳንዱ የስኳር በሽታ ታካሚዎች ይሰበስባል። እባክዎ ለእያንዳንዱ ጥያቄ ተገቢውን ምላሽ ይስጡ **።**

| **ክፍል አንድ ፡ የማህበራዊ እና ስነ-ሕዝብ ባህሪያት ጋር የተያያዙ መጠይቆች** | | | | | | | | | | | | | | |
| --- | --- | --- | --- | --- | --- | --- | --- | --- | --- | --- | --- | --- | --- | --- |
| SNO | ጥያቄዎች | መልስ | | | | | ኮዶች | | | | መዝለል | አስተያየቶች | | |
| 101 | ፆታ | ወንድ | | | | | 1 | | | |  |  | | |
|  |  | ሴት | | | | | 2 | | | |  |  |  |  |
| 102 | ዕድሜ | ----------------- | | | | |  | | | |  |  | | |
| 103 | የጋብቻ ሁኔታ | ያላገባ | | | | | 1 | | | |  |  | | |
|  |  | ያገባ | | | | | 2 | | | |  |  |  |  |
|  |  | መበለት | | | | | 3 | | | |  |  |  |  |
|  |  | ፍቺ/ተለያይቷል | | | | | 4 | | | |  |  |  |  |
| 104 | የትምህርት ደረጃ | ያልተማረ | | | | | 1 | | | |  |  | | |
|  |  | ማንበብ እና መፃፍ የሚችል | | | | | 2 | | | |  |  |  |  |
|  |  | የመጀመሪያ ደረጃ ትምህርት የተማረ | | | | | 3 | | | |  |  |  |  |
|  |  | ሁለተኛ ደረጃ ትምህርት እና ከዚያ በላይ | | | | | 4 | | | |  |  |  |  |
| 105 | የስራ ሁኔታ | ሥራ ፈላጊ | | | | | 1 | | | |  |  | | |
|  |  | የመንግስት ሰራተኛ | | | | | 2 | | | |  |  |  |  |
|  |  | የግል ድርጅት ተቀጥሮ የሚሰራ | | | | | 3 | | | |  |  |  |  |
|  |  | አርሶ አደር | | | | | 4 | | | |  |  |  |  |
|  |  | ነጋዴ | | | | | 5 | | | |  |  |  |  |
|  |  | ሌሎች……………………………. | | | | |  | | | |  |  |  |  |
| 106 | የመኖሪያ ቦታ | ከተማ | | | | | 1 | | | |  |  | | |
|  |  | ገጠር | | | | | 2 | | | |  |  |  |  |
| **ክፍል ሁለት፡ የስኳር በሽታ ጋር የተያያዙ የጭንቀት መለኪያ (DDS-17) መጠይቆች** | | | | | | | | | | | | | | |
| ከዚህ ቀጥሎ የቀረቡት እያንዳንዳቸው ባለፈው ወር ውስጥ በምን ያህል መጠን ጭንቀት ውስጥ ሆነው እንደነበር የሚያሳዩ መጠይቆች ናቸው፡፡ የሚስማሙበትን መልስ **(√)** ምልክት በማድረግ ያረጋግጡ | | | | | | | | | | | | | | |
|  | | ችግር  አይደለም (1) | | አነስተኛ ችግር (2) | | መካከለኛ ችግር (3) | | መጠነኛ ችግር (4) | | | ከባድ ችግር (5) | | በጣም ከባድ ችግር (6) | |
| 201 | የስኳር ህመም በየቀኑ አዕምሮየን እና አካላዊ አቅሜን ከመጠን በላይ እያዳከመ እንደሆነ ይሰማኛል |  | |  | |  | |  | | |  | |  | |
| 202 | ሐኪሜ ስለ ስኳር በሽታ እና የስኳር በሽታን መቆጣጠሪያ መንገዶች በቂ እውቀት እንደሌለው ይሰማኛል |  | |  | |  | |  | | |  | |  | |
| 203 | ከስኳር በሽታ ጋር ስለመኖር ሳስብ ቁጣ፣ ፍርሃት እና/ወይም የመንፈስ ጭንቀት ይሰማኛል |  | |  | |  | |  | | |  | |  | |
| 204 | ሐኪሜ የስኳር በሽታዬን እንዴት መቆጣጠር እንዳለብኝ በቂ ግንዛቤ እንደማይሰጠኝ ይሰማኛል |  | |  | |  | |  | | |  | |  | |
| 205 | በደም ውስጥ ያለውን የስኳር መጠን በበቂ ሁኔታ እየለካሁ እንዳልሆነ ይሰማኛል |  | |  | |  | |  | | |  | |  | |
| 206 | በስኳር ህመም ልማዴ ውስጥ ብዙ ጊዜ እንደወደቅኩ ይሰማኛል |  | |  | |  | |  | | |  | |  | |
| 207 | ጓደኞቼ ወይም ቤተሰብ እኔን ለመንከባከብ በቂ ድጋፍ እንደማይሰጡ ይሰማኛል (ለምሳሌ፣ ከፕሮግራሜ ጋር የሚቃረኑ ተግባራትን ማቀድ፣ “የተሳሳተ” ምግብ እንድበላ ማበረታታት) |  | |  | |  | |  | | |  | |  | |
| 208 | የስኳር በሽታ ሕይወቴን እንደተቆጣጠረው ይሰማኛል |  | |  | |  | |  | | |  | |  | |
| 209 | ሀኪሜ ጭንቀቴን/ ሃሳቤን በበቂ ሁኔታ ትኩረት እንደማይሰጠው ይሰማኛል |  | |  | |  | |  | | |  | |  | |
| 210 | የስኳር በሽታን ለመቆጣጠር በየቀኑ በራስ የመተማመን ስሜት አልፈጠረልኝም |  | |  | |  | |  | | |  | |  | |
| 211 | ምንም ባደርግ መጨረሻ ላይ የረዥም ጊዜ ውስብስብ እንደሚገጥመኝ ይሰማኛል |  | |  | |  | |  | | |  | |  | |
| 212 | ከጥሩ የምግብ እቅድ ጋር በበቂ ሁኔታ ትስስር እንዳልፈጠርኩ ይሰማኛል |  | |  | |  | |  | | |  | |  | |
| 213 | ጓደኞች ወይም ቤተሰብ ከስኳር በሽታ ጋር መኖር ምን ያህል ከባድ እንደሆነ እንደማይረዱ ይሰማኛል |  | |  | |  | |  | | |  | |  | |
| 214 | ከስኳር በሽታ ጋር መኖር ከመጠን በላይ የመጨናነቅ ስሜት ፈጥሮብኛል |  | |  | |  | |  | | |  | |  | |
| 215 | የስኳር ህመምተኛ መሆኔ አዘውትሬ የማገኘው ዶክተር የለኝም የሚል ስሜት ይሰማኛል |  | |  | |  | |  | | |  | |  | |
| 216 | የስኳር በሽተኛ በመሆኔ እራሴን ለማስተዳደር ፍላጎት የለኝም |  | |  | |  | |  | | |  | |  | |
| 217 | ጓደኞች ወይም ቤተሰቦች የምፈልገውን ስሜታዊ ድጋፍ እንደማይሰጡኝ ይሰማኛል |  | |  | |  | |  | | |  | |  | |
| **ክፍል ሦስት ሀ ፡ ከበሽታው ጋር የተያያዙ መጠይቆች** | | | | | | | | | | | | | | |
| 301 | ከስኳር በሽታ ጋር ምን ያህል ጊዜ ኖርክ/ሽ | ……………… | | | | |  | | | |  |  | | |
| 302 | የስኳር በሽታ ተጓዳኝ ውስብስብነት አለበዎት? | አዎ | | | | | 1 | | | |  |  | | |
|  |  | የለም | | | | | 2 | | | |  |  |  |  |
| 303 | አዎ ከሆነ በሽተኛው የትኛው አይነት ውስብስብ ነው | ኔፍሮፓቲ(ከስኳር በሽታ ጋር የተያያዘ የኩላሊት ህመም/በሽታ) | | | | | 1 | | | |  |  | | |
|  |  | ሬቲኖፓቲ (ከስኳር በሽታ ጋር የተያያዘ የዐይን ህመም/በሽታ) | | | | | 2 | | | |  |  |  |  |
|  |  | የስኳር በሽታ እግር ቁስለት | | | | | 3 | | | |  |  |  |  |
|  |  | ሌሎች………………… | | | | |  | | | |  |  |  |  |
| 304 | ባለፉት 3 ወራት ውስጥ የስኳር መጠን በደም ውስጥ መቀነስ ክስተት አጋጥሞዎታል | አዎ | | | | | 1 | | | |  |  | | |
|  |  | የለም | | | | | 2 | | | |  |  |  |  |
| 305 | በአሁኑ ጊዜ ምን ዓይነት መድሃኒት ነው የሚወስዱት ***(ከአንድ በላይ አማራጭ መምረጥ ይቻላል)*** | በአፍ | | | | | 1 | | | |  |  | | |
|  |  | ኢንሱሊን(መርፌ) | | | | | 2 | | | |  |  |  |  |
|  |  | የአፍ እና ኢንሱሊን(መርፌ) | | | | | 3 | | | |  |  |  |  |
| **የታካሚ ጤና መጠይቅ (PHQ-9) በመጠቀም የድብርት መጠንን መለኪያ ጥያቄዎች** | | | | | | | | | | | | | | |
|  | ባለፉት 2 ሳምንታት ውስጥ፣ የሚከተሉት ችግሮች ለምን ያህል ቀናት እንዳስቸገሩዎት **(“”)** በመጠቀም መልስዎን ያመልክቱ | | | | | | | | | | | | | |
| 306 | ጥያቄዎች | አልቸገረም(0) | | | አብዛኛውን ቀናት (1) | | | | ከግማሽ ቀናቶች በላይ (2) | | | | | በየቀኑ (3) |
| 307 | ነገሮችን ለመስራት አነስተኛ ፍላጎት አለኝ |  | | |  | | | |  | | | | |  |
| 308 | የድብርት ወይም የተስፋ መቁረጥ ስሜት አለኝ |  | | |  | | | |  | | | | |  |
| 309 | የእንቅልፍ ማጣት ወይም ብዙ መተኛት ስሜት አለኝ |  | | |  | | | |  | | | | |  |
| 310 | የድካም ወይም ጉልበት ማነስ/መድከም ስሜት አለኝ |  | | |  | | | |  | | | | |  |
| 311 | የምግብ ፍላጎት መቀነስ ወይም ከመጠን በላይ መብላት እፈልጋለሁ |  | | |  | | | |  | | | | |  |
| 312 | ስለራስዎ መጥፎ ስሜት መሰማት ወይም እራስህን እና ቤተሰብህን እየጎዳህ እንደሆነ ይሰማሀል |  | | |  | | | |  | | | | |  |
| 313 | እንደ ጋዜጣ ማንበብ ወይም ቴሌቪዥን መመልከት ባሉ ነገሮች ላይ ትኩረት ማጣት |  | | |  | | | |  | | | | |  |
| 314 | ሌሎች ሰዎች ሊገነዘቡት በማይችሉት መንቀሳቀስ ወይም መናገር፤ ወይም በተቃራኒው በጣም ጨካኝ ወይም እረፍት የሌለበት ከመሆኑ የተነሳ ከወትሮው በበለጠ ብዙ መንቀሳቀስ ስሜት አለኝ |  | | |  | | | |  | | | | |  |
| 315 | ብትሞት ይሻላል ወይም እራስህን ብትጎዳ ይሻልሃል የሚሉ ሃሳቦች መምጣት ይታይብኛል |  | | |  | | | |  | | | | |  |
| **ክፍል ሦስት ለ ፡ የታካሚዎችን የህክምና ካርድ በመገምገም የሚሞሉ ከበሽታው ጋር የተያያዙ መጠይቆች** | | | | | | | | | | | | | | |
| 316 | አሁን ያለው የጾም የደም ስኳር መጠን (FBG) (mg/dl) | | …………………………. | | | |  | | | |  |  | | |
| 317 | የታካሚው ሲስቶሊክ የደም ግፊት መጠን(mmHg) | | ---- | | | |  | | | |  |  | | |
| 318 | የታካሚው ዲያስቶሊክ የደም ግፊት መጠን(mmHg) | | …………………………. | | | |  | | | |  |  | | |
| 319 | የታካሚው ቁመት (በሜትር) | | …………………………. | | | |  | | | |  |  | | |
| 320 | የታካሚው ክብደት (ኪ.ግ.) | | …………………………. | | | |  | | | |  |  | | |
| 321 | የስኳር በሽታ ዓይነት | | ዓይነት 1 | | | | 1 | | | |  |  | | |
|  |  |  | ዓይነት 2 | | | | 2 | | | |  |  |  |  |
| **ክፍል አራት፡ ከታካሚው ስነ-ባህሪ ጋር የተያያዙ መጠይቆች** | | | | | | | | | | | | | | |
| 401 | የአካል ብቃት እንቅስቃሴ መርሃ ግብር አዘጋጅተዋል | የለም | | | | | | | | 1 |  |  | | |
|  |  | በሳምንት አንዴ/ሁለት ጊዜ | | | | | | | | 2 |  |  |  |  |
|  |  | መደበኛ የአካል ብቃት እንቅስቃሴ (>ሶስት ጊዜ) | | | | | | | | 4 |  |  |  |  |
| 402 | አማካይ የእንቅልፍ ሰአት | ………………………… | | | | | | | |  |  |  | | |
| ሱስ የሚያሲዙ ንጥረ ነገሮች/ዕፅ አጠቃቀምን መገምገሚያ መጠይቅ | | | | | | | | | | | | | | |
| በህይወትዎ ውስጥ ከሚከተሉት ንጥረ ነገሮች (ሱስ የሚያሲዙ ዕፆች ) ውስጥ የትኛውን ተጠቅመህ ታውቃለህ/ሽ? | | | | | | | | | | | | | | |
| 403 | የትምባሆ ምርቶች (ሲጋራ፣ ጫት፣ ወዘተ ) | አዎ | | | | | | | | 1 |  |  | | |
|  |  | የለም | | | | | | | | 2 |  |  |  |  |
| 404 | የአልኮል መጠጦች (ቢራ, ወይን፤ወዘተ) | አዎ | | | | | | | | 1 |  |  | | |
|  |  | የለም | | | | | | | | 2 |  |  |  |  |
| 405 | ካናቢስ (ማሪዋና፣ ሳር፣ ሃሽሽ) | አዎ | | | | | | | | 1 |  |  | | |
|  |  | የለም | | | | | | | | 2 |  |  |  |  |
| 406 | ኮኬይን (ኮክ) | አዎ | | | | | | | | 1 |  |  | | |
|  |  | የለም | | | | | | | | 2 |  |  |  |  |
| 407 | የአምፌታሚን(አእምሮን አነቃቂ መድሃኒቶች) | አዎ | | | | | | | | 1 |  |  | | |
|  |  | የለም | | | | | | | | 2 |  |  |  |  |
| 408 | በመተንፈሻ አካላት የሚወሰዱ (ናይትረስ፣ ቤንዚን፣ ቀለም ማቅጠኛ፣ወዘተ) | አዎ | | | | | | | | 1 |  |  | | |
|  |  | የለም | | | | | | | | 2 |  |  |  |  |
| 409 | የእንቅልፍ ክኒን (ዲያዜፓም፣ አልፕራዞላም፣ ፍሉኒቲያዜፓም ፣ ሚዳዞላም፣ ወዘተ) | አዎ | | | | | | | | 1 |  |  | | |
|  |  | የለም | | | | | | | | 2 |  |  |  |  |
| 410 | ሃሉሲኖጅንስ ( እንጉዳይ, ኬታሚን, ወዘተ ) | አዎ | | | | | | | | 1 |  |  | | |
|  |  | የለም | | | | | | | | 2 |  |  |  |  |
| 411 | ከባድ ህመም ማስታገሻወች(ሄሮይን ፣ ሞርፊን ፣ ሜታዶን ፣ ቡፕረኖርፊን፣ወዘተ) | አዎ | | | | | | | | 1 |  |  | | |
|  |  | የለም | | | | | | | | 2 |  |  |  |  |
| 412 | ሌላ ካለ- ይግለጹ | …………………. | | | | | | | |  |  |  | | |
| 413 | ከላይ ላለው ንጥረ ነገር "አዎ" ከሆነ መልስዎ፣ ባለፉት ሶስት ወራት ውስጥ የጠቀሷቸውን ንጥረ ነገሮች ለምን ያህል ጊዜ ተጠቅመዋል? | የለም | | | | | | | | 0 |  |  | | |
|  |  | አንዴ ወይም ሁለቴ | | | | | | | | 2 |  |  |  |  |
|  |  | ወርሃዊ (በወር 1-3 ጊዜ) | | | | | | | | 3 |  |  |  |  |
|  |  | በየሳምንቱ (በሳምንት 1-4 ጊዜ) | | | | | | | | 4 |  |  |  |  |
|  |  | በየቀኑ (በሳምንት 5-7 ጊዜ) | | | | | | | | 6 |  |  |  |  |
| 414 | ከላይ ላለው ንጥረ ነገር/ዕፅ "አዎ" ከሆነ መልስዎ፣ ባለፉት ሶስት ወራት ውስጥ ምን ያህል ጊዜ ለመጠቀም ከፍተኛ ፍላጎት ነበረዎት። | የለም | | | | | | | | 0 |  |  | | |
|  |  | አንዴ ወይም ሁለቴ | | | | | | | | 3 |  |  |  |  |
|  |  | ወርሃዊ (በወር 1-3 ጊዜ) | | | | | | | | 4 |  |  |  |  |
|  |  | በየሳምንቱ (በሳምንት 1-4 ጊዜ) | | | | | | | | 5 |  |  |  |  |
|  |  | በየቀኑ (በሳምንት 5-7 ጊዜ) | | | | | | | | 6 |  |  |  |  |
| 415 | ከላይ ላለው ንጥረ ነገር/ዕፅ "አዎ" ከሆነ መልስዎ፣ ባለፉት ሦስት ወራት ውስጥ፣ የዚህ ንጥረ ነገር /ዕፅ አጠቃቀምዎ ምን ያህል የጤና፣ ማህበራዊ፣ ህጋዊ ወይም የገንዘብ ችግር ዳረገዎት። | የለም | | | | | | | | 0 |  |  | | |
|  |  | አንዴ ወይም ሁለቴ | | | | | | | | 4 |  |  |  |  |
|  |  | ወርሃዊ (በወር 1-3 ጊዜ) | | | | | | | | 5 |  |  |  |  |
|  |  | በየሳምንቱ (በሳምንት 1-4 ጊዜ) | | | | | | | | 6 |  |  |  |  |
|  |  | በየቀኑ (በሳምንት 5-7 ጊዜ) | | | | | | | | 7 |  |  |  |  |
| 416 | ከላይ ላለው ንጥረ ነገር/ዕፅ "አዎ" ከሆነ መልስዎ፣ ባለፉት ሶስት ወራት ውስጥ፣ በዚህ ንጥረ ነገር/ዕፅ አጠቃቀምዎ ምክንያት ምን ያህል ጊዜ ከእርስዎ የሚጠበቀውን ተግባር ሳያደርጉ ቀሩ። | የለም | | | | | | | | 0 |  |  | | |
|  |  | አንዴ ወይም ሁለቴ | | | | | | | | 5 |  |  |  |  |
|  |  | ወርሃዊ (በወር 1-3 ጊዜ) | | | | | | | | 6 |  |  |  |  |
|  |  | በየሳምንቱ (በሳምንት 1-4 ጊዜ) | | | | | | | | 7 |  |  |  |  |
|  |  | በየቀኑ (በሳምንት 5-7 ጊዜ) | | | | | | | | 8 |  |  |  |  |
| 417 | ከላይ ላለው ንጥረ ነገር/ዕፅ "አዎ" ከሆነ መልስዎ፣ ጓደኛዎ ወይም ዘመድዎ ወይም ሌላ ማንኛውም ሰው ስለ እርስዎ የንጥረ ነገር/ዕፅ አጠቃቀም ስጋት ገልጾ አያውቅም። | የለም | | | | | | | | 0 |  |  | | |
|  |  | አዎ "ባለፉት 3 ወራት ውስጥ ነው" | | | | | | | | 6 |  |  |  |  |
|  |  | አዎ “ግን ባለፉት 3 ወራት ውስጥ አይደለም” | | | | | | | | 3 |  |  |  |  |
| 418 | ከላይ ላለው ንጥረ ነገር/ዕፅ "አዎ" ከሆነ መልስዎ፣ የአደንዛዥ ንጥረ ነገር /ዕፅ አጠቃቀምን ለመቀነስ ሞክረው ነገር ግን ሳይሳካልዎት ያውቃል። | የለም | | | | | | | | 0 |  |  | | |
|  |  | አዎ "ባለፉት 3 ወራት ውስጥ ነው" | | | | | | | | 6 |  |  |  |  |
|  |  | አዎ “ግን ባለፉት 3 ወራት ውስጥ አይደለም” | | | | | | | | 3 |  |  |  |  |
| **የኦስሎ ማህበራዊ ድጋፍ ልኬት መጠይቆች** | | | | | | | | | | | | | | |
| 414 | ምን ያህል ሰዎች ናቸው ለእርስዎ በጣም ቅርብ ሆነው ትልቅ የግል ችግር ካጋጠመዎት በእነሱ ላይ የሚተማመኑት/የሚያማክሩት | የለም | | | | | | | | 1 |  |  | | |
|  |  | "1-2" | | | | | | | | 2 |  |  |  |  |
|  |  | "3-5" | | | | | | | | 3 |  |  |  |  |
|  |  | ≥ 5 | | | | | | | | 4 |  |  |  |  |
| 415 | ሰዎች በምታደርገው ነገር ምን ያህል ፍላጎት እና አሳቢነት ያሳያሉ | ምንም | | | | | | | | 1 |  |  | | |
|  |  | ትንሽ | | | | | | | | 2 |  |  |  |  |
|  |  | እርግጠኛ አደለሁም | | | | | | | | 3 |  |  |  |  |
|  |  | አንዳንድ | | | | | | | | 4 |  |  |  |  |
|  |  | ብዙ | | | | | | | | 5 |  |  |  |  |
| 416 | ባስፈለገዎት ጊዜ ከጎረቤት ተግባራዊ እርዳታ ማግኘት ምን ያህል ቀላል ነው | በጣም ከባድ | | | | | | | | 1 |  |  | | |
|  |  | አስቸጋሪ | | | | | | | | 2 |  |  |  |  |
|  |  | ይቻላል | | | | | | | | 3 |  |  |  |  |
|  |  | ቀላል | | | | | | | | 4 |  |  | | |
|  |  | በጣም ቀላል | | | | | | | | 5 |  |  |  |  |

**አመሰግናለሁ!**
