# Supplementary material for: Diabetes-related distress and associated factors among adult diabetes mellitus patients attending public hospitals in Gedio zone, southern Ethiopia: Mediation analysis
Source: PLoS One. 2025 Sep 29;20(9):e0331655. doi: 10.1371/journal.pone.0331655 (PMC12478905; doi:10.1371/journal.pone.0331655)
Supplement: S3 Questionnaires — (DOCX) [file pone.0331655.s003.docx]

**Gedeufatika qola**

Hordofoleyetika tarja woraqata

eyyendinexxi hordofoleye

Nageange? Anni ----------------------------------hiyemanon dilixxe universitena’n fayuitetixe sayinisexxe kolleje gido qo’ne meisa’nexxe baratole tukese’e kaba afemenen. Qo’ne uda’nexxi sukarixxe dhukuliye’ni sukarinile dhukubin abidendexxe rakuwa’ni kada woda unverisitena udake hospitaluwana’ni eet alifinenen. Tinni qo’ne sukarixxe dhibon abidendexxa rako garigari hisate’e garigaran. Wele ba’laxxi qo’nuwana bogi gido fayunitetixxi huje heregate’e keinisha erigatan. Tene’a qo’ne wo’mitexxe kadasha ha’noki kiphina garigarisi yo’osha hasisan. Tene qo’ne gido ha’noxxi hordofe haranga haraniga eyya ha’noxen erikatexxen.tene qo’ne gido hordofa yokan hordfa gopha hospital gido alifitina’eke garigarisa’ni mite fugona gesisabaxxe kada wodda hene yanana qoritumuwa hissa gopha dandetinan.qo’ne tene gido keake qoritumuwia utinaki hisichuwi maqo heqemeke kada wodda ha’noki summi hanona kokesemaban. Etan kini utinaki hisichi ayunte ha’noxxin welit abidemaban. Qo’ne tene’n hanona qorituma afinole hene yana’nina yado buki asake Manji gami’ni ilite qo’nisanjo affa dandesisan.

Immi’ni kulemeke tarijisha qo’nete’ni hordifate’e eet hitia;ne?

Eet---------------------------------------jamari wawot---------------------------- Galatefatanon!

Hisicho uwa’nwkiki besisa --------------------

Keito tariji miyaziye 2024 kena’n genbote 2024 miti mitinge sukarike dhukubixxe dhukuloyena’n bukki asendan. tenea mite mitinige qoritumuwia hasisaka hisicho uwwe.

| **Kuta mite mittuntettixxana aradinxxe arddan woltti abidendexxa qorrtumwaa** | | | | | | | | | | | | | | |
| --- | --- | --- | --- | --- | --- | --- | --- | --- | --- | --- | --- | --- | --- | --- |
| **lakosa** | **qortumuwa** | **hisicho** | | | | | **Garigari hisajo** | | | | **xoga** | **yada** | | |
| 101 | Laba meyute | labaka | | | | | 1 | | | |  |  | | |
|  |  | meyaxxa | | | | | 2 | | | |  |  |  |  |
| 102 | Woga | --------------------- | | | | |  | | | |  |  | | |
| 103 | Adhatikana herumi jeja | Adhebake/xxe | | | | | 1 | | | |  |  | | |
|  |  | Adheke/xxe | | | | | 2 | | | |  |  |  |  |
|  |  | Minika anna afebaxxe | | | | | 3 | | | |  |  |  |  |
|  |  | tikexxe | | | | | 4 | | | |  |  |  |  |
| 104 | Barchotika jeja | Barateke/xxe | | | | | 1 | | | |  |  | | |
|  |  | Nababana boresa dandeake/xxe | | | | | 2 | | | |  |  |  |  |
|  |  | Takaka kobo barateke/xxe | | | | | 3 | | | |  |  |  |  |
|  |  | Langaka kobin ima barateke/xxe | | | | | 4 | | | |  |  |  |  |
| 105 | Hujetika jeja | Huje harisake | | | | | 1 | | | |  |  | | |
|  |  | Mutumatika hujalo | | | | | 2 | | | |  |  |  |  |
|  |  | Manjiba hujake | | | | | 3 | | | |  |  |  |  |
|  |  | wisalo | | | | | 4 | | | |  |  |  |  |
|  |  | dadalito | | | | | 5 | | | |  |  |  |  |
|  |  | Welena ---------------- | | | | | 6 | | | |  |  |  |  |
| 106 | Hedhatika loli | qacha | | | | | 1 | | | |  |  | | |
|  |  | hadigido | | | | | 2 | | | |  |  |  |  |
| **Kuta lame sukarike dhukubin kexexa ellobaxxe hedinoke kilama (DDS-17) qoritumuwa** | | | | | | | | | | | | | | |
| Saeke ajenji gido hone qico bojamesexxa lelishaka qoritumuwa eet hisicho**(√)** besisin kokesi | | | | | | | | | | | | | | |
|  | | Rakoteban rako(1) | | Sholaxxa rako (2) | | Odite rako (3) | | Shixoxa rako (4) | | | Jabaxa rako (5) | | Haraniga jabaxxa rako (6) | |
| 201 | Sukarixxi dhibo barabaranin hedho atixxa gophisanen |  | |  | |  | |  | | |  | |  | |
| 202 | Ogeshi anki sukara fachefatatika oran wo’ma ege’na afebasha laba’nen |  | |  | |  | |  | | |  | |  | |
| 203 | Sukarixxa dhinon hedhati hedano woda mufena soda’ana yado laqisenda’en |  | |  | |  | |  | | |  | |  | |
| 204 | Ogesichi dhukuba anka hita fachefatanoxxa wo’ma yada uwa’nebacho laba’nen |  | |  | |  | |  | | |  | |  | |
| 205 | Munde anxi gido hedheka sukara ada mukisedhabe’nosha laba’nen |  | |  | |  | |  | | |  | |  | |
| 206 | Surarixxi dhibo baracho gigo ebnesha laba’nen |  | |  | |  | |  | | |  | |  | |
| 207 | Hadinan/jaluwi ane’e kokisha’nebacho laba’nen fakenake’e juje anixin weli labaxxa yane herega hasisabaxxa sagale etenosha jajabesa |  | |  | |  | |  | | |  | |  | |
| 208 | Sukarixxi dhibo hedho anixxa ducha abidesha laba’nen |  | |  | |  | |  | | |  | |  | |
| 209 | Ogesichi hagdawi/heduma anike yada uwa’nebacho laba’nen |  | |  | |  | |  | | |  | |  | |
| 210 | Sukara anika fachefatate’e ifi’ni adatatixxa yaddo uwa’nebasha laba’nen |  | |  | |  | |  | | |  | |  | |
| 211 | Mchona labole muxamixe yana’n mami geyaxxa laqisenda’nen |  | |  | |  | |  | | |  | |  | |
| 212 | Elloxxa ette udaxxa elloki/wo’mi yane abidenidebasha ege’nisha’nen |  | |  | |  | |  | | |  | |  | |
| 213 | Jaluwi/hadinan sukarixxe dhibon hedha jabaxxe kadexxa henda’nebacho laba’nen |  | |  | |  | |  | | |  | |  | |
| 214 | Sukarixxe dhinoni hedha jaba qicini hojogemanosha asita’nen |  | |  | |  | |  | | |  | |  | |
| 215 | Sukarike dhukulicha kade’e duchi woda afeneki ogesich/dokiterich hedha’neban hiyye hedanon |  | |  | |  | |  | | |  | |  | |
| 216 | Sukarike dhukulicha kade’e iffi galichate’e eyya afebo’non |  | |  | |  | |  | | |  | |  | |
| 217 | Hadinan/jaluwi hasenexxa eyya una’nebacho laba’nen |  | |  | |  | |  | | |  | |  | |
| **Kuta sase 1, dhukubiki welit affemeka qortumuwa** | | | | | | | | | | | | | | |
| 301 | Sukarike dhukubin hite qico turutete? | ……………………………. | | | | |  | | | |  |  | | |
| 302 | Sukarixxe dhibon wliti shiqaxi mekiya hexxe? | eet | | | | | 1 | | | |  |  | | |
|  |  | wawot | | | | | 2 | | | |  |  |  |  |
| 303 | Eet kadole hene meki? | Kalateteixxe dhibo | | | | | 1 | | | |  |  | | |
|  |  | Elletixxi dhibo | | | | | 2 | | | |  |  |  |  |
|  |  | Lekatixxi dhibo/madoa | | | | | 3 | | | |  |  |  |  |
|  |  | welenna | | | | | 4 | | | |  |  |  |  |
| 304 | Sase agenji gido sukar munde gido xe’yatik jeji hedhe? | Eet | | | | | 1 | | | |  |  | | |
|  |  | wawot | | | | | 2 | | | |  |  |  |  |
| 305 | Ta’axxeyana’ni hene kutaa qorisa adhitate? | Affoike | | | | | 1 | | | |  |  | | |
|  |  | Marifeti/insuline | | | | | 2 | | | |  |  |  |  |
|  |  | Afoikina/marifeti/insuline | | | | | 3 | | | |  |  |  |  |
| **Hedhdtike jejixxa jijira(dhukulichixxe fayuniteka laqito(PHQ-9) laofatat qajibotixxe mikeka qortumuwa** | | | | | | | | | | | | | | |
| **saxexe lame torba gido kaba hunda hexexi rakuwa hitexa barra rakisexxa laofatat hisicho kokesi** | | | | | | | | | | | | | | |
|  | Qoritumuwa | Rakatebo’nin (0) | | | Yo’oxxa barra (1) | | | | Qame baruwa imma(2) | | | | | Arabaranide’n (3) |
| 306 | Yanuwa hujate’e haniqo yorte afenen |  | | |  | | | |  | | | | |  |
| 307 | Qajibo/ade woxxa laqisenda’nen |  | | |  | | | |  | | | | |  |
| 308 | Mugo gopha/bacha muga laqisenda’nen |  | | |  | | | |  | | | | |  |
| 309 | Gonphe/huna gopha hexxen |  | | |  | | | |  | | | | |  |
| 310 | Etatixe yoritexxi xe’ya/birra sae eeta hsanen |  | | |  | | | |  | | | | |  |
| 311 | Ifi’nina /hadinanake’ni ellobaxxa hedio hedana ifi’ni bala gesisa laqisenida’nen |  | | |  | | | |  | | | | |  |
| 312 | Gazexxa nababa/tv udatisha hexxexe yane’ni hubacho uwwa gopha laqisenda’nen |  | | |  | | | |  | | | | |  |
| 313 | Weli Manji heda dandeaba qico’ni lele’ma/hasoa yokan dayefacho hexexe kadexena’n kexexa edi immi cala lele’ma affenen |  | | |  | | | |  | | | | |  |
| 314 | Retetele woya /ifi yakitetele woyya hiyaki hedumi lela’nen |  | | |  | | | |  | | | | |  |
| **Kuta sase 2, dhukubaleyetixxa ci’lotixxa karide memekisat wonishendaxxa dhukubiki abidendexxa qo’nuwa** | | | | | | | | | | | | | | |
| 315 | Ta’a hexxexi xometixxi/eteboxong mundetixxi gulucose (FBG) (mg/dl) | | ……………………… | | | |  | | | |  |  | | |
| 316 | Dhukulichinixxi imixxi mundetixxi dhaba (mmHg) | | …………………… | | | |  | | | |  |  | | |
| 317 | Dhukulicninixxi butixi mundetixxi dhaba (mmHg) | | ……………………. | | | |  | | | |  |  | | |
| 318 | Dhukulichinxi qerin metiret | | ……………………….. | | | |  | | | |  |  | | |
| 319 | Dhukulichink auri | | ……………………….. | | | |  | | | |  |  | | |
| 320 | Surarike dhukubixxa bifa | | Bifa1 | | | |  | | | |  |  | | |
|  |  |  | Bifa2 | | | |  | | | |  |  |  |  |
| **Kuta shole, dhukubinke jejin afemeka qortumuwa** | | | | | | | | | | | | | | |
| 401 | Bisinke jabenixxs lele’ma asate’e gisitete? | Hedheban | | | | | | | | 1 |  |  | | |
|  |  | Torbate’me mitele/lamele | | | | | | | | 2 |  |  |  |  |
|  |  | Detite bisinxxa lele’ma >sasele | | | | | | | | 3 |  |  |  |  |
| 402 | Hedinotixi muginixxi yana(seate) | ---------------------- | | | | | | | |  |  |  | | |
| **Suse abidisaxxa yane laofatatike memekisatika qorte** | | | | | | | | | | | | | | |
| **hedhotixxe yanan’ni buti’n hexexa suse abidisaxxa hene yane laofate egendete** | | | | | | | | | | | | | | |
| 403 | Tanbotixxa gumuwa/sigara/cate | Eet | | | | | | | | 1 |  |  | | |
|  |  | Hedheban | | | | | | | | 2 |  |  |  |  |
| 404 | Alikole ha’wa ( bira,woyine welina eetin | Eet | | | | | | | | 1 |  |  | | |
|  |  | Hedheban | | | | | | | | 2 |  |  |  |  |
| 405 | Kanabise(mariwana,hashishe | Eet | | | | | | | | 1 |  |  | | |
|  |  | Hedheban | | | | | | | | 2 |  |  |  |  |
| 413406 | Kokiyene(koke) | Eet | | | | | | | | 1 |  |  | | |
|  |  | Hedheban | | | | | | | | 2 |  |  |  |  |
| 407 | Enifitamine(samo danasaka qorisuwa | Eet | | | | | | | | 1 |  |  | | |
|  |  | Hedheban | | | | | | | | 2 |  |  |  |  |
| 408 | Hafura adhatike bisia adhinaka(nayitires,bezila,welena/etan | Eet | | | | | | | | 1 |  |  | | |
|  |  | Hedheban | | | | | | | | 2 |  |  |  |  |
| 409 | Muginxxa kinine (diazepam,aliprazolam,midazolam welna eten | Eet | | | | | | | | 1 |  |  | | |
|  |  | Hedheban | | | | | | | | 2 |  |  |  |  |
| 410 | Halusinginese(ketamine welena etan | Eet | | | | | | | | 1 |  |  | | |
|  |  | Hedheban | | | | | | | | 2 |  |  |  |  |
| 411 | Jabaxxa dhibo urisaxxa( heroine,morphine welena etan | Eet | | | | | | | | 1 |  |  | | |
|  |  | Hedheban | | | | | | | | 2 |  |  |  |  |
| 412 | Welina hedhole kuli | ----------------------------- | | | | | | | |  |  |  | | |
| 413 | Imi’ni hexexa guma yane /etse eet kadole hisichi saeke sase agenji gido hone qico laofateta’a eya afeta’a/ | hedheban | | | | | | | | 0 |  |  | | |
|  |  | Mitele/lamele | | | | | | | | 2 |  |  |  |  |
|  |  | Agenjoke’n (1—3 yana) | | | | | | | | 3 |  |  |  |  |
|  |  | Torbate’n (1-4yana) | | | | | | | | 4 |  |  |  |  |
|  |  | Barabaranide’n torbate’n (5-7yana) | | | | | | | | 6 |  |  |  |  |
| 414 | Imi’ni hexexa guma yane /etse eet kadole hisichi saeke sase agenji gido hone qico laefatate’e lumoxxa eyunte afete | hedheban | | | | | | | | 0 |  |  | | |
|  |  | Mitele/lamele | | | | | | | | 3 |  |  |  |  |
|  |  | Agenjoke’n (1—3 yana) | | | | | | | | 4 |  |  |  |  |
|  |  | Torbate’n (1-4yana) | | | | | | | | 5 |  |  |  |  |
|  |  | Barabaranide’n torbate’n (5-7yana) | | | | | | | | 6 |  |  |  |  |
| 415 | Imi’n ke’eke yada’ni eet kadole hisich saeke sase agenji gido kone yada’n laofatate’e hone qico fayunte mituntena mituma malaqi hone qico rakise | hedheban | | | | | | | | 0 |  |  | | |
|  |  | Mitele/lamele | | | | | | | | 4 |  |  |  |  |
|  |  | Agenjoke’n (1—3 yana) | | | | | | | | 5 |  |  |  |  |
|  |  | Torbate’n (1-4yana) | | | | | | | | 6 |  |  |  |  |
|  |  | Barabaranide’n torbate’n (5-7yana) | | | | | | | | 7 |  |  |  |  |
| 416 | Imi’ni hedheke yada’n eet kadole hisich saeke sase agenji gido kone yada’n laofata hone qico asitinebang gate’e | hedheban | | | | | | | | 0 |  |  | | |
|  |  | Mitele/lamele | | | | | | | | 5 |  |  |  |  |
|  |  | Agenjoke’n (1—3 yana) | | | | | | | | 6 |  |  |  |  |
|  |  | Torbate’n (1-4yana) | | | | | | | | 7 |  |  |  |  |
|  |  | Barabaranide’n torbate’n (5-7yana) | | | | | | | | 8 |  |  |  |  |
| 417 | Imi’ni hedheke yada’n eet kadole hisich jaluwi/hadinan/welmjanji atia yada yane laofatate soda’a shiqise ege’ne? | hexxeban | | | | | | | | 0 |  |  | | |
|  |  | Eet saeke sase agenji gido | | | | | | | | 6 |  |  |  |  |
|  |  | Eet saeke sase agenji edo | | | | | | | | 3 |  |  |  |  |
| 418 | Imi’ni hedheke yada’n eet kadole hisich biso dilesitaxxa yane xeishate’e hede’na’a kadebangi gate’e egende? | hexxeban | | | | | | | | 0 |  |  | | |
|  |  | Eet saeke sase agenji gido | | | | | | | | 6 |  |  |  |  |
|  |  | Eet saeke sase agenji edo | | | | | | | | 3 |  |  |  |  |
| **Osilese ardinxxe kiphixxa mikixxa qophe** | | | | | | | | | | | | | | |
| 419 | Hite mana ate’e rakko gelitole shuqakina adatinak yokan malichisexinak | Hedheban | | | | | | | | 1 |  |  | | |
|  |  | 1-2 | | | | | | | | 2 |  |  |  |  |
|  |  | 3-5 | | | | | | | | 3 |  |  |  |  |
|  |  | >5 | | | | | | | | 4 |  |  |  |  |
| 420 | Hujatoxxe yana Manji hone qico eyatana heda lelisha’ne | Hedheban | | | | | | | | 1 |  |  | | |
|  |  | Shixoki | | | | | | | | 2 |  |  |  |  |
|  |  | Moshi | | | | | | | | 3 |  |  |  |  |
|  |  | Miti miti | | | | | | | | 4 |  |  |  |  |
|  |  | Yo’oki | | | | | | | | 5 |  |  |  |  |
| 421 | Hasisaxxa yana gargarisa ola’akena’n alifate’e hone qico sholaxxe | Haranga jabaxxe | | | | | | | | 1 |  |  | | |
|  |  | Rakisaxxe | | | | | | | | 2 |  |  |  |  |
|  |  | Dandesisan | | | | | | | | 3 |  |  |  |  |
|  |  | sholaxxe | | | | | | | | 4 |  |  | | |
|  |  | Haranga sholaxxe | | | | | | | | 5 |  |  |  |  |

Galatefatanon!
