# Supplementary material for: Diabetes-related distress and associated factors among adult diabetes mellitus patients attending public hospitals in Gedio zone, southern Ethiopia: Mediation analysis
Source: PLoS One. 2025 Sep 29;20(9):e0331655. doi: 10.1371/journal.pone.0331655 (PMC12478905; doi:10.1371/journal.pone.0331655)
Supplement: Attachment — Submitted filename: Reviewer.docx [file pone.0331655.s005.docx]

Title: Diabetic-related distress and associated factors among adult diabetes mellitus patients
attending public hospitals in Gedio zone, Southern Ethiopia

Thank you for the opportunity to review this manuscript.

- I respectfully recommend a thorough review of the manuscript for language and grammar to improve clarity and readability.
- Please specify the type of diabetes in the title. At the end of the introduction, the authors mention “like type 1 diabetes mellitus,” but there is no reference to the type of diabetes in the methods section.
- The authors wrote: “The sample size was determined by using the previous study variable of HbA1c (percentage of outcome in the unexposed group = 93.2% and adjusted odds ratio = 5.49).” Please add a reference to this section.
- Please explain the phrase “fulfilled the inclusion criteria.”
- Reference the validity and reliability of the questionnaires used in the target population.
- Mention how physical activity was assessed.
- The researchers wrote: “Data was collected by eight BSc degree nurses through face-to-face interviews, self-administered questionnaires, and reviewing the patient charts depending on the occasion.” Please specify which questionnaire was collected using each of the mentioned methods.
- In this section, for data quality assurance, please mention the Cronbach’s alpha for each questionnaire you have assessed.
- The authors stated that approximately one-sixth (16.6% or 84) of the sample were unable to read and write. How were the questionnaires completed for these individuals?
- The sample size was 521 individuals, and ultimately 506 were included. Please mention the reasons for excluding individuals.
- The authors stated that they used the PHQ to measure social support; please provide a reference that this has been done previously. A four-point Likert scale of PHQ-9 and three items from the Oslo social support scale were also used to assess depression and participants’ level of social support.
- In Table 4, based on which variables are the adjustments reported?
- Severe depression is also significant in Table 4, but it is not addressed in the discussion section.
