# Supplementary material for: Diabetes-related distress and associated factors among adult diabetes mellitus patients attending public hospitals in Gedio zone, southern Ethiopia: Mediation analysis
Source: PLoS One. 2025 Sep 29;20(9):e0331655. doi: 10.1371/journal.pone.0331655 (PMC12478905; doi:10.1371/journal.pone.0331655)
Supplement: Attachment — Submitted filename: Response to reviewers.docx [file pone.0331655.s007.docx]

**RESPONSE LETTER FOR COMMENTS**

**August 16, 2025**

First of all, we want to express our deepest gratitude to all reviewers and editorials for their detailed and thoughtful suggestions and for allowing us to resubmit a revised version of our PLOS ONE manuscript PONE-D-25-10454- Diabetic-related distress and associated factors among adult diabetes mellitus patients attending public hospitals in Gedio zone, Southern Ethiopia. In accordance with your previous message, we are resubmitting the revised manuscript with some modifications by the stated deadline of August 17, 2025.We have incorporated the recommended changes in the manuscript to the best of our ability and we feel that our revised manuscript is significantly improved as a result of your advice. In addition to this, we have responded specifically to each suggestion and have highlighted it in the manuscript and response letter.

Thank you once again for your time and attention to our manuscript.

**Editorial comments and response**

1. Please ensure that your manuscript meets PLOS ONE's style requirements, including those for file naming. The PLOS ONE style templates can be found at <https://journals.plos.org/plosone/s/file?id=wjVg/PLOSOne_formatting_sample_main_body.pdf> and<https://journals.plos.org/plosone/s/file?id=ba62/PLOSOne_formatting_sample_title_authors_affiliations.pdf>

**Response #1:** Thank you for your comment. We have made some modifications in accordance with the journal’s guidelines

2. Thank you for stating the following financial disclosure: “Dilla University fund this study”

Please state what role the funders took in the study. If the funders had no role, please state: "The funders had no role in study design, data collection and analysis, decision to publish, or preparation of the manuscript." If this statement is not correct you must amend it as needed. Please include this amended Role of Funder statement in your cover letter; we will change the online submission form on your behalf.

**Response #2:** Thank you for your comment. It has been incorporated into the cover letter**.**

3. When completing the data availability statement of the submission form, you indicated that you will make your data available on acceptance. We strongly recommend all authors decide on a data sharing plan before acceptance, as the process can be lengthy and hold up publication timelines. Please note that, though access restrictions are acceptable now, your entire data will need to be made freely accessible if your manuscript is accepted for publication. This policy applies to all data except where public deposition would breach compliance with the protocol approved by your research ethics board. If you are unable to adhere to our open data policy, please kindly revise your statement to explain your reasoning and we will seek the editor's input on an exemption. Please be assured that, once you have provided your new statement, the assessment of your exemption will not hold up the peer review process.

**Response #3:** Thank you for your comment. We have uploaded the data as supporting information in the system.

4. [Please remove all personal information, ensure that the data shared are in accordance with participant consent, and re-upload a fully anonymized data set.
   Note: spreadsheet columns with personal information must be removed and not hidden as all hidden columns will appear in the published file.
   Additional guidance on preparing raw data for publication can be found in our Data Policy (https://journals.plos.org/plosone/s/data-availability#loc-human-research-participant-data-and-other-sensitive-data) and in the following article: http://www.bmj.com/content/340/bmj.c181.long.](Please%20remove%20all%20personal%20information,%20ensure%20that%20the%20data%20shared%20are%20in%20accordance%20with%20participant%20consent,%20and%20re-upload%20a%20fully%20anonymized%20data%20set.Note:%20spreadsheet%20columns%20with%20personal%20information%20must%20be%20removed%20and%20not%20hidden%20as%20all%20hidden%20columns%20will%20appear%20in%20the%20published%20file.Additional%20guidance%20on%20preparing%20raw%20data%20for%20publication%20can%20be%20found%20in%20our%20Data%20Policy%20(https://journals.plos.org/plosone/s/data-availability#loc-human-research-participant-data-and-other-sensitive-data) and in the following article: http://www.bmj.com/content/340/bmj.c181.long.)

**Response #4:** Thank you for your comment. We have revised and re-uploaded the anonymized dataset.

5. more about the validity and reliability ( psychometry ) of employed questionnaires in your study in local language.

**Response #5:** Thank you for your constructive comment. The reliability and validity of the questionnaire were incorporated under the 'data quality assurance' section.

6. This study needs mediation analysis for robust conclusion.

**Response #6:** Thank you for your constructive feedback. We conducted a mediation analysis for this study and identified a variable that met the criteria to be considered a mediating variable. A detailed description of the analysis is provided in the 'Data Analysis' section, and the findings are reported in the 'Results' section of the study.

**Reviewer’s comments response**

**Reviewer's Responses to Questions**

1. Is the manuscript technically sound, and do the data support the conclusions?

**Reviewer response #1:** The manuscript must describe a technically sound piece of scientific research with data that supports the conclusions. Experiments must have been conducted rigorously, with appropriate controls, replication, and sample sizes. The conclusions must be drawn appropriately based on the data presented.

Reviewer #1: Partly

Reviewer #2: Yes

**Author's response #1:** Thank you for your constructive suggestion. Based on your feedback, we have revised our conclusion to align with the dataset. Regarding your recommendation to conduct an experimental study, we have included it as a suggestion for future researchers to undertake an intervention-based study.

2. Has the statistical analysis been performed appropriately and rigorously?

**Reviewer response #2:** Reviewer #1: Yes

Reviewer #2: Yes

**Author's** **Response #2:** Thank you for your response

3. Have the authors made all data underlying the findings in their manuscript fully available?

**Reviewer response #3:** The [PLOS Data policy](http://track.editorialmanager.com/CL0/http:%2F%2Fwww.plosone.org%2Fstatic%2Fpolicies.action%23sharing/1/010f0197cfd59712-02f69e0d-3a5c-4a31-a89d-561e38d074fd-000000/3HkXW3G9RTsfLzTRzojgAH8DSM4aiGzKxNwsUF7oJsE=218) requires authors to make all data underlying the findings described in their manuscript fully available without restriction, with rare exception (please refer to the Data Availability Statement in the manuscript PDF file). The data should be provided as part of the manuscript or its supporting information, or deposited to a public repository. For example, in addition to summary statistics, the data points behind means, medians and variance measures should be available. If there are restrictions on publicly sharing data—e.g. participant privacy or use of data from a third party—those must be specified.

**Author's Response #3:** Thank you for your comments. All data described in the manuscript have been provided as supporting information

4. Is the manuscript presented in an intelligible fashion and written in standard English?

**Reviewer response #4:** PLOS ONE does not copyedit accepted manuscripts, so the language in submitted articles must be clear, correct, and unambiguous. Any typographical or grammatical errors should be corrected at revision, so please note any specific errors here.

**Author's Response #4:** Thank you for your comment. We have corrected the spelling and grammatical errors within the manuscript.

**Reviewer #1 comments and responses**

1. I respectfully recommend a thorough review of the manuscript for language and grammar to improve clarity and readability.

**Response #1**: Thank you for your feedback. We have made corrections to the grammatical and language errors.

2. Please specify the type of diabetes in the title. At the end of the introduction, the authors mention “like type 1 diabetes mellitus,” but there is no reference to the type of diabetes in the methods section.

**Response #2:** Thank you for your comment. We revised the introduction to indicate that the study focuses on type 1 and type 2 diabetes mellitus. In addition, we stated at the operational definition that the study specifically focuses on these two types of diabetes.

3. The authors wrote: “The sample size was determined by using the previous study variable of HbA1c (percentage of outcome in the unexposed group = 93.2% and adjusted odds ratio = 5.49).” Please add a reference to this section.

**Response #3:** Thank you for your comment. We have added a reference for the sample size at the end of the adjusted odds ratio for the study variable (HbA1c).

4. Please explain the phrase “fulfilled the inclusion criteria.”

**Response #4:** Thank you for your comments. The list inclusion criteria that need to be fulfilled were stated under a separate heading titled “inclusion and exclusion criteria” in the method part of the study.

5. Reference the validity and reliability of the questionnaires used in the target population

**Response #5:** Thank you for your constructive comment. The validity and reliability (internal consistency) were addressed in the “Data quality assurance” part of the study.

6. Mention how physical activity was assessed.

**Response #6:** Thank you for your comment. Physical activity was categorized in the table 3 section as “‘never’, ‘once/twice a week’, and ‘regular exercise (more than three times a week)”.

7. The researchers wrote: “Data was collected by eight BSc degree nurses through face-to-face interviews, self-administered questionnaires, and reviewing the patient charts depending on the occasion.” Please specify which questionnaire was collected using each of the mentioned methods.

**Response #7**: Thank you for your comment. It is incorporated at the end of “Data collection tools.

8. In this section, for data quality assurance, please mention the Cronbach’s alpha for each questionnaire you have assessed.

**Response #8:** Thank you for your comment. We calculated the Cronbach's alpha for the scale data items assessed in the questionnaires. This has been stated in the data quality assurance section

9. The authors stated that approximately one-sixth (16.6% or 84) of the sample were unable to read and write. How were the questionnaires completed for these individuals?

**Response #9:** Thank you for your comment. This is the reason we decided to use two methods for administering the questionnaire: self-administered forms and face-to-face interviews. We applied a face-to-face interview to the patient who could not read or write.

10. The sample size was 521 individuals, and ultimately 506 were included. Please mention the reasons for excluding individuals.

**Response #10:** Thank you for raising this concern. These respondents were not intentionally excluded; they met the inclusion criteria, but, due to their busy schedules and being in a hurry to get to work, they were unwilling to complete the questionnaire and were therefore recorded as non-respondents. This reason is mentioned in the “limitations of the study” section.

11. The authors stated that they used the PHQ to measure social support; please provide a reference that this has been done previously. A four-point Likert scale of PHQ-9 and three items from the Oslo social support scale were also used to assess depression and participants’ level of social support.

**Response #11:** Thank you for your comment. References were added to the indicated part.

12. In Table 4, based on which variables are the adjustments reported?

**Response #12:** Thank you for your comment. In the description section, we included the total number of variables assessed in the study, stating that out of twenty-one variables, thirteen had a p-value less than 0.25. These variables were considered candidates for multivariable ordinal logistic regression. A p-value less than 0.05 was considered a significant predictor of diabetes-related distress. In Table 4, a footnote explains the abbreviations used: AOR stands for adjusted odds ratio, COR for crude odds ratio, * indicates p < 0.25, and ** indicates p < 0.05.

13. Severe depression is also significant in Table 4, but it is not addressed in the discussion section.

**Response #13:** Thank you for your comment. Based on the footnote description of Table 4, depression is one of the variables that fulfilled the criteria for binary ordinal logistic regression, which is a p value less than 0.25, indicated as * in the footnote but in multivariable ordinal logistic regression, the p value is not less than 0.05 for this variable which is stated ** in the footnote. So, since the p-value is not less than 0.05, we conclude that depression is not a predictor of diabetic related distress.

**Reviewer #2 comments and responses**

1. The text needs some grammatical editing.

**Response #1:** Thank you for your comment. We have made corrections to the sentences that required grammatical editing for this study.

2. Keywords: Please provide keywords from the MeSH database.

**Response #2:** Thank you for your constructive comment. We provide keywords based on the MeSH database

3. The authors mentioned that “all data are fully available without restriction” in their Data Availability statement, but I couldn’t find it in the review portal.

**Response #3:** Thank you for your feedback. The data were submitted as supportive material.

4. Please change “diabetic-related” to “diabetes-related”.

**Response #4:** Thank you for your comment. We have corrected it based on your suggestion.

5. Clearly mention the study design (e.g., cross-sectional) in the title.

**Response #5:** Thank you for your comment**.** We placed the analysis (mediation analysis) next to the title instead of the study design to highlight that additional analyses were conducted.

6. Abbreviations used in the text (e.g., aOR) must be written in complete form in their first use.

**Response #6:** Thank you for your comment. We have corrected it based on your suggestion.

7. In the methods section, please mention the data collection tools (e.g., scales and questionnaires), study period, and eligibility criteria instead of the statistical software.

**Response #7:** Thank you for your comment. We have corrected it based on your suggestion.

8. You can remove the software names from the methodology.

**Response #8:** Thank you for your comment. We have corrected it based on your suggestion.

9. Please include the final analyzed sample size in the Results section.

**Response #9:** Thank you for your comment. We have corrected it based on your suggestion.

10. Line 38: Please describe which complications.

**Response #10:** Thank you for your comment. In our study, the specific “type of complications” was not significant; only the presence and absence of complications showed a significant association.

11. Nearly all of the citations are irrelevant or from secondary sources. Please double-check all the citations and ensure they support the cited text.

**Response #11:** Thank you for your constructive comment. We replaced the secondary source citation with the primary (original) source.

12. Lines 73-79: I believe it is better to move this paragraph to the discussion section.

**Response #12:** Thank you for your comment; we have incorporated it into the discussion section (lines 313–317) based on your suggestion.

13. Line 92: Please cite the CSA 2011 report.

**Response #13:** Thank you for your comment. we have made the necessary changes and provided a citation.

14. Line 99: Please mention that this region is in Southern Ethiopia.

**Response #14:** Thank you for your comment. A correction has been made based on your comment (line 96).

15. Population: How did you exclude the patients with a history of distress before the DM onset (to make sure that the distress is completely associated with the DM)? If not excluded, please mention this as a limitation of the study.

**Response #15:** Thank you for your constructive comments: we included this as one of the limitations of the study.

16. Line 104: How did you define the “critically ill”?

**Response #16:** Thank you for your comment. We modified it as “unable to communicate,” which indicates the patient needs intensive monitoring due to their critical health condition.

17. Lines 108-109: Please cite the previous study used for sample size calculation.

**Response #17:** Thank you for your comment. We include a citation based on your recommendation.

18. Lines 111-120: The text is somehow vague and hard to understand, and needs to be clearer.

**Response #18:** Thank you for your Supportive feedback. We have revised it to improve the readability of the content.

19. Study variables: You can remove this section since important variables are discussed in the following text.

**Response #19:** Thank you for your comment. Based on your recommendation, it has been excluded from the study.

20. Again, citations in the Methods section are irrelevant or poorly support the cited text. Please double-check and make the necessary amendments.

**Response #20:** Thank you for your comment. We have made revisions to the citation section in the operational definitions and in the data collection tool part.

21. Line 132: How was a patient considered to have DM? Which criteria were used? Please describe it clearly and in detail. If medical records were used, then please specify each hospital’s criteria.

**Response #21:** Thank you for your comment. As described at the end of the “Data Collection Tool” section of the manuscript, chart reviews of medical records were undertaken for selected clinical data components, among which “type of diabetes” was included. so determination of whether a patient had diabetes mellitus (type 1 or type 2) was based on the information documented in their medical records. The criteria applied by the hospitals to consider DM were based on the patient's FBG level. The exact level the hospital used was stated in the manuscript.

22. Lines 147-148: Please cite the reference for the DDS-17 scale.

**Response #22:** Thank you for your comment. We have included a citation for the scale in the manuscript as per your recommendation.

23. Line 169: Do you mean written informed consent by “written and signed permission letters”?

**Response #23:** Thank you for your constructive feedback. This is to indicate that written permission was obtained from each hospital to conduct this study. Modifications were made to improve the clarity of the sentence and the overall paragraph.

24. . Line 173: Please change to “To minimize disruption to patient care, participants were approached only after their medical treatment was completed.”

**Response #24:** Thank you for your comment. The manuscript has been modified in accordance with your suggestion.

25. Please consider reporting the frequency percents up to one decimal point.

**Response #25:** Thank you for your comment. The percentages presented in the frequency table are already reported to one decimal place.

26. Table 2: Based on the Methods section, patients were only included if they were diagnosed in the last 3 months. The information presented in the “Duration of living with diabetes” row is not consistent with the mentioned eligibility criteria.

**Response #26:** Thank you for your comment. The inclusion criteria state that diabetic patients who had been under follow-up for at least three months and were not diagnosed within the past three months were eligible. Therefore, patients with a follow-up period exceeding three months were qualified to participate in the study.

27. Table 2: Please merge the “Diabetic associated complications” and “Type of complications” rows.

**Response #27:** Thank you for your comment. These two variables are different. The first variable, diabetic complication status, indicates whether the patient has any complications. If a patient is reported as “yes” for having complications, the next variable identifies the specific type of complication they experienced. Since these variables are completely independent and measure different aspects, it is difficult to incorporate one another.

28. Table 2: Please mention the score/value range for each subcategory of the “Depression” and “BMI” rows.

**Response #28:** Thank you for your comment. We incorporated your suggestion into both variables.

29. Please edit the “bivariate” to “univariate” throughout your text.

**Response #29:** Thank you for your comment. The Univariate analysis does not examine the relationship between variables, particularly between independent and outcome variables, but bivariable regression analyzes each independent variable with the outcome variable separately. So we believe that making this change is not suitable for this study.

30. Please edit the COR to simply OR and AOR to aOR.

**Response #30:** Thank you for your comment. It has been corrected based on your recommendation.

31. Table 4: Please add a p-value column for the crude ORs.

**Response #31:** Thank you for your comment. It has been added next to the 95%CI of OR

32. Table 4: Please indicate the rows with OR or aOR equal to one are reference subcategories.

**Response #32:** Thank you for your comment**.** We renamed OR or AOR values equal to one as ‘Reference.

33. Please report the 95% confidence interval for the 11.46%.

**Response #33:** Thank you for your comment. We incorporated it based on your recommendation

34. Limitations: Please also mention that since all of the participants were selected from the hospital patients, this can lead to a selection bias because of including the patients with more severe DM.

**Response #34:** Thank you for your comment. We have made the modifications based on your suggestions.

**Note:** As the questions in the Word document were similar to those uploaded in the system, responses were addressed collectively.
